# Pan-cancer integrative analyses dissect the remodeling of endothelial cells in human cancers

**DOI:** 10.1093/nsr/nwae231

**Published:** 2024-07-11

**Authors:** Jinhu Li, Dongfang Wang, Fei Tang, Xinnan Ling, Wenjie Zhang, Zemin Zhang

**Affiliations:** Biomedical Pioneering Innovation Center (BIOPIC), School of Life Sciences, Peking University, Beijing 100871, China; Biomedical Pioneering Innovation Center (BIOPIC), School of Life Sciences, Peking University, Beijing 100871, China; Biomedical Pioneering Innovation Center (BIOPIC), School of Life Sciences, Peking University, Beijing 100871, China; Academy for Advanced Interdisciplinary Studies, Peking University, Beijing 100871, China; Biomedical Pioneering Innovation Center (BIOPIC), School of Life Sciences, Peking University, Beijing 100871, China; Biomedical Pioneering Innovation Center (BIOPIC), School of Life Sciences, Peking University, Beijing 100871, China; Biomedical Pioneering Innovation Center (BIOPIC), School of Life Sciences, Peking University, Beijing 100871, China; Academy for Advanced Interdisciplinary Studies, Peking University, Beijing 100871, China

**Keywords:** tumor endothelial cell, pan-cancer, single-cell RNA-seq, immunotherapy

## Abstract

Therapeutics targeting tumor endothelial cells (TECs) have been explored for decades, with only suboptimal efficacy achieved, partly due to an insufficient understanding of the TEC heterogeneity across cancer patients. We integrated single-cell RNA-seq data of 575 cancer patients from 19 solid tumor types, comprehensively charting the TEC phenotypic diversities. Our analyses uncovered underappreciated compositional and functional heterogeneity in TECs from a pan-cancer perspective. Two subsets, *CXCR4*^+^ tip cells and *SELE*^+^ veins, represented the prominent angiogenic and proinflammatory phenotypes of TECs, respectively. They exhibited distinct spatial organization patterns, and compared to adjacent non-tumor tissues, tumor tissue showed an increased prevalence of *CXCR4*^+^ tip cells, yet with *SELE*^+^ veins depleted. Such functional and spatial characteristics underlie their differential associations with the response of anti-angiogenic therapies and immunotherapies. Our integrative resources and findings open new avenues to understand and clinically intervene in the tumor vasculature.

## Teaser

Using integrated scRNA-seq data, this study uncovers the compositional and functional heterogeneity of tumor endothelial cells, highlighting the angiogenic *CXCR4*^+^ tip cells and proinflammatory SELE+ veins, and their relationship with different treatments.

## INTRODUCTION

Perturbing the tumor microenvironment (TME) rather than only cancer cells underpins the success of immunotherapies [1,2]. Although the primary targets of immunotherapies are immune cells, mounting evidence suggests that other TME components exert profound impacts on the progression of tumors and treatment efficacy [3,4]. In particular, endothelial cells (ECs), constituting the inner lining of the tumor vasculature, have been unequivocally proven to play crucial roles in cancer development [5–7]. Accordingly, endeavors to pursue therapeutic strategies targeting tumor endothelial cells (TECs), or tumor angiogenesis, have been performed [8]; however, while certain have been clinically approved, most anti-angiogenic therapies (AATs) only achieve limited efficacy in clinical settings [4,6,9]. Alternative vascular targeting strategies, such as normalizing or reprogramming TECs, have also been explored, yet few promising improvements have been made [10,11]. Resolving these puzzles warrants a deep understanding of the biology and clinical relevance of TECs.

Multifaceted roles of TECs, apart from the conventional focus on their function of oxygen and nutrient supply to promote cancer development, are increasingly unraveled [12–14]. For instance, TECs engage in extracellular matrix (ECM) organization and remodeling, and they can secret cytokines to mediate anti-tumor immune responses [4,15]. The aberrant tumor vasculature could also hinder T-cell recruitment and induce inferior T-cell infiltration [13,16,17]. On the other hand, emerging studies have emphasized that TECs may represent an underappreciated heterogeneous population and harbor diverse characteristics among different cancers [18,19]. Within the endothelium in tumors, tip cells are regarded as primary targets of AATs; however, these cells occupy a relatively low proportion of TECs in certain cancer types and their systematic comparison across cancers is absent [18,20,21]. In addition, while the roles of other TEC components have also been uncovered, their functions and heterogeneity are largely unexplored [18,19]. It is thus needed to comprehensively investigate the composition and phenotype of TECs as well as their distinctions across different cancers.

Single-cell RNA-seq (scRNA-seq) technologies empower the high-resolution characterization of cellular heterogeneity in the TME, bringing new opportunities to illuminate the spectrum of TECs [18,22,23]. A study of human lung cancers has discovered multiple previously unexplored phenotypes of TECs [20]. In prostate cancers, the TEC-associated CXCR4-CXCL12 crosstalk has been identified as a novel AAT target [24]. However, for most scRNA-seq studies of human TMEs, it is still challenging to sufficiently explore the heterogeneity of ECs, primarily because they are relatively rare in tumors. In addition, while the organ specificity of ECs has been depicted in mice through integrative single-cell analyses [25,26], such systematic interrogation is lacking in humans. Certain preliminary comparative analyses of TECs have been conducted, yet the limited number of cells and cancer types examined could potentially obscure the identification of TEC heterogeneity [27,28]. Together, these inspired us to conduct a large-scale single-cell analysis of TECs in human cancers.

Herein, we collected scRNA-seq datasets from 19 major solid tumor types, obtaining ∼150 000 high-quality ECs. A comprehensive pan-cancer EC atlas was then established, with the population structure of TECs charted. We elucidated the phenotypical characteristics of fine-grained EC subsets and uncovered their compositional and functional alterations from adjacent non-tumor to tumor tissues. Importantly, we highlighted distinct TEC characteristics among various patients and cancer types, especially in terms of two representative statuses of TEC phenotype, proinflammatory and angiogenic, which could impact the treatment efficacy of AAT and immune checkpoint blockade (ICB). Our analyses greatly expand the understanding of TEC properties in human cancers and these data present a rich resource for promoting the development of therapeutic strategies targeting TECs.

## RESULTS

### Landscape of pan-cancer endothelial cells at single-cell resolution

**Figure 1.**
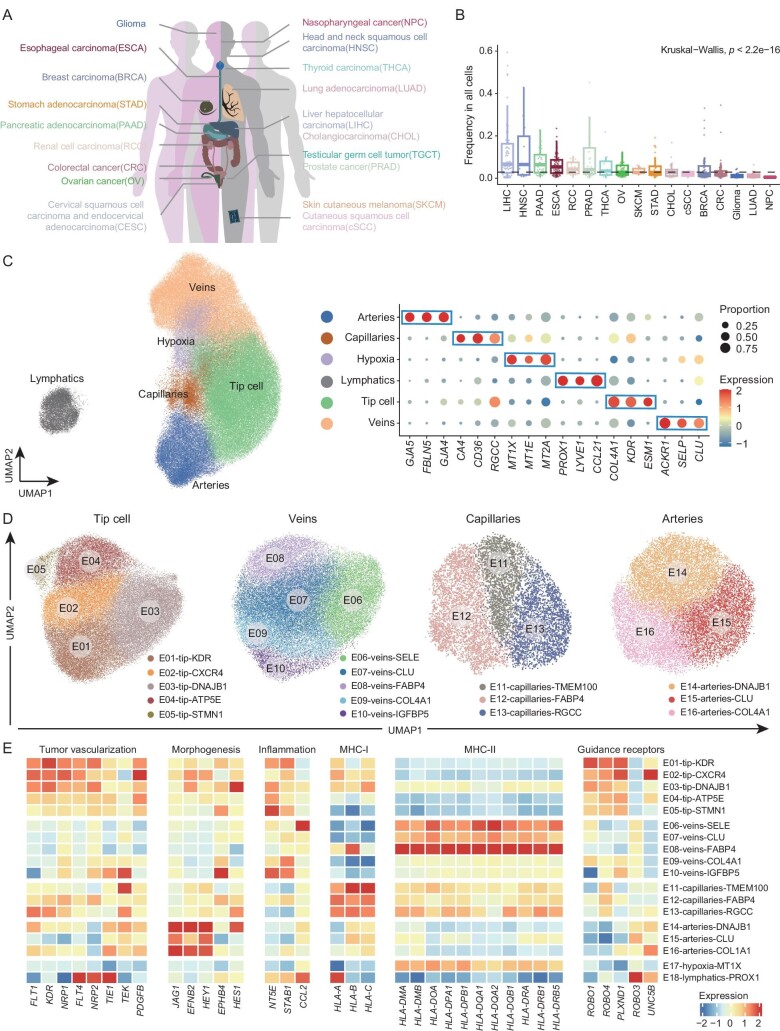
Construction of a pan-cancer single-cell atlas of endothelial cells. (A) The 19 cancer types involved in the integrative pan-cancer analysis. (B) The frequency of ECs within all cells for tumor samples across various cancer types. Dashed line indicates the median value. Kruskal–Wallis test. (C) Uniform Manifold Approximation and Projection (UMAP) visualization of the major compartments of ECs (left), and bubble heatmap showing the expression patterns of corresponding signature genes of each major compartment (right). Dot size represents the proportion of expressing cells. Color indicates the Z score scaled gene expression levels. (D) UMAP visualizations of all EC subsets within their corresponding major compartments. (E) Heatmap showing the expression patterns of functional genes in EC subsets. Color indicates the Z score scaled gene expression levels.

We assembled scRNA-seq datasets for 19 major solid tumor types to comprehensively dissect the population structure and functional alteration of ECs within tumors (Fig. 1A; Table S1). All samples collected have not undergone any prior cell sorting that might shift the intrinsic cellular frequencies. After stringent quality control and extracting ECs through unsupervised clustering in each dataset, we achieved a final collection of 149 854 high-quality ECs, covering 723 tumor or adjacent non-tumor samples from 575 patients (Fig. S1A–D; Supplementary Methods). Typically, the proportion of ECs within all TME cellular components was relatively low, with a median value of 2.8% across all analyzed tumor samples (Fig. 1B). The abundance of TECs varied dramatically among different cancer types. In LIHC, TECs occupied 6.6% of all cellular components, while in NPC, only ∼0.5% were TECs. The relative scarcity of TECs makes it challenging to systematically investigate their fine-grained heterogeneity in a single study. Our large-scale collection of ECs, instead, provides a unique opportunity to deeply explore TEC characteristics.

To unbiasedly delineate the population structure of ECs at the pan-cancer level, we integrated these high-quality cells and systematically performed two rounds of unsupervised clustering (Fig. S1E; Supplementary Methods). During the first round, major compartments, including one group of lymphatic ECs and multiple groups of blood vascular ECs—arteries, veins, capillaries, and tip cells, were identified (Fig. 1C; Table S2). As expected, lymphatic ECs, specifically expressing markers such as *PROX1, LYVE1*, and *CCL21*, exhibited obvious transcriptional differences with those blood vascular ECs. Apart from these compartments, another EC group, with a heightened expression of hypoxia- and oxidative stress-associated genes, such as *MT1X, MT1E*, and *MT2A* [29], were distinguished from others. Interestingly, although detected in a wide variety of tumors, these cells were enriched in certain cancers including PRAD and RCC (Fig. S1F), indicating that they might be influenced by cancer type- or organ-associated factors.

The second round of unsupervised clustering was performed within each major compartment separately to identify fine-grained subpopulation structures (Figs 1D and S1G). Notably, a series of subsets were discerned, with each subset characterized by the high expression of their specific signature genes within the major compartment (Table S2). In particular, tip cells could be divided into five subsets, with E01 featuring the highest expression of *KDR*. E02 exhibited a relatively higher expression of the receptor genes of *CXCL12* including *CXCR4* and *ACKR3*. The other three cell states were associated with stress responses (E03), ATP synthesis (E04), and cell cycles (E05), respectively (Fig. S1H). For veins, five subsets were detected. Intriguingly, the expression of *CLU* and collagen-associated genes, including *COL4A1* and *COL4A2*, demonstrated opposite trends in these subsets (Fig. S1I), potentially representing non-overlapping phenotypes of veins in the TME. Notably, E-selectin (*SELE*) was specially expressed in E06, and only E08 had an obvious high expression of *FABP4*. Capillary subset E12 also highly expressed this gene. We discerned another two capillary subsets, E11 and E13, with the former uniquely expressing genes like *TMEM100* and *HPGD* (Fig. S1J). Finally, within the arteries, E15 and E16 showed opposite expression trends of *CLU* and collagen-associated genes, while E14 resembled the activated status of E03, featuring a high expression of stress response-associated genes (Figs S1K and S2A). Briefly, our integrative atlas enabled a high-resolution depiction of the transcriptional phenotype of ECs at the pan-cancer level.

### Transcriptome heterogeneity of distinct endothelial cell subsets

Multifaceted functional roles can be exerted by ECs during cancer progression. We next examined whether these functions were shared by distinct subsets or attributed to specific groups. First, various functional aspects pertinent to tumor vascularization were interrogated. We observed that multiple receptor tyrosine kinases (RTKs), such as *FLT1, KDR*, and *NRP1*, were widely expressed in tip cell subsets, while other RTK genes including *FLT4, NRP2*, and *TIE1*, demonstrated the highest expression level in lymphatic ECs (E18), consistent with previous studies [19,30]. *PDGFB*, a gene related to pericyte recruitment and angiogenesis [7,30], was also highly expressed in tip cell subsets (Fig. 1E). All artery subsets, E14, E15, and E16, exhibited conserved expression of genes involved in arterial morphogenesis, such as *JAG1* and *EFNB2* [31,32]; by contrast, *EPHB4*, associated with venous morphogenesis [30,33], was highly expressed in one vein subset, E10.

Our high-resolution atlas provided additional insights into other reported EC functions. EC subsets expressed genes encoding factors of inflammatory responses and antigen presentation (Fig. 1E). For instance, E06-veins-SELE was characterized by the high expression of *CCL2*, capable of recruiting various immune cells [34], indicating its potential proinflammatory role. In contrast, tip cell subsets exhibited the high expression of *NT5E*, reported to mitigate effector T-cell homing, and *STAB1*, which can enhance Treg recruitment [12,19,35,36]. In addition, E06, together with E07 and E08, all with a weak expression of collagen-associated genes, highly expressed MHC-II relevant genes. A previous lung cancer study has defined a subset of scavenging capillaries associated with antigen presentation [20]. In our atlas, E11-capillaries-TMEM100 resembled the transcriptional characteristics of this subset, for example, highly expressing complement-relevant genes (Fig. S2B). While E11 exhibited a modest expression of MHC-II relevant genes, it highly expressed a series of MHC-I relevant genes (Fig. 1E). These results demonstrated that distinct EC subsets could engage in diverse intratumoral immune responses. Previous studies have found that tip cells could express guidance receptors, potentially analogizing the function of the axonal growth cone [7,37]. We found tip cells highly expressing various axon guidance receptors, but with a diverse pattern among different subsets (Fig. 1E). Taken together, the calibrated fine-grained subsets through the large-scale scRNA-seq analysis charted the underappreciated functional heterogeneity of TECs.

### The phenotypical axes of endothelial cells in human cancers

**Figure 2.**
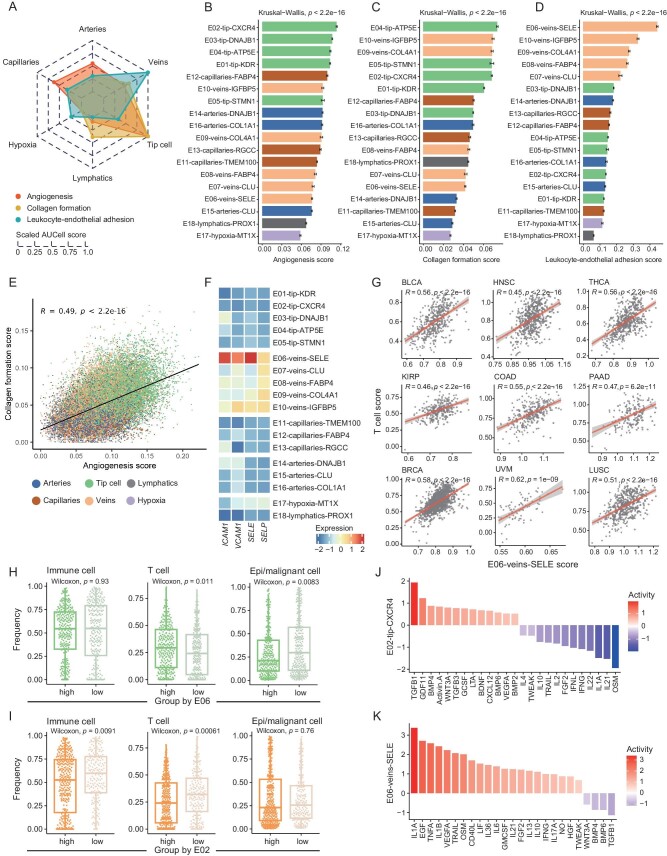
Phenotypical heterogeneity of endothelial cell subsets. (A) Radar chart comparing the functional scores of all major compartments of ECs. (B–D) Bar plots comparing the AUCell scores of angiogenesis (B), collagen formation (C) and leukocyte-endothelial adhesion (D), among all EC subsets. Kruskal–Wallis test. Data presented as mean ± s.e.m. (E) Scatterplot showing the correlation of angiogenesis score with collagen formation score in all ECs. Each dot represents a single cell. Pearson correlation test. (F) Heatmap showing the expression patterns of *ICAM1, VCAM1, SELE* and *SELP*, among all EC subsets. Color indicates the Z score scaled gene expression levels. (G) Scatterplot showing the correlations of E06-veins-SELE score with T cell score in each cancer type of TCGA dataset. Each dot represents a tumor sample. Pearson correlation test. (H and I) Boxplots illustrate the relationship between the frequencies of E06-veins-SELE (H) and E02-tip-CXCR4 (I) and those of other cell types in the TME. Two-sided unpaired Wilcoxon test. (J and K) The predicted cytokine activities, calculated by Cytosig, in E02-tip-CXCR4 cells (J) and E06-veins-SELE cells (K).

To untangle the phenotypical divergence of different EC subsets, we focused on three functional axes of tumor vasculature, including angiogenesis, collagen formation, and leukocyte-endothelial adhesion (Supplementary Methods). Consistent with the high expression of multiple RTKs, tip cells exhibited the highest angiogenesis score (Fig. 2A). Except for E05-tip-STMN1, all tip cell subsets harbored higher angiogenesis activities than other subsets, with the highest level detected in E02-tip-CXCR4 (Fig. 2B). We further examined angiogenesis activities in each cancer type, observing that the prominent angiogenesis potential of E02-tip-CXCR4 was conserved in almost all analyzed cancers (Fig. S2D). Consistently, compared with other tip cell subsets, E02 upregulated genes involved in pathways such as blood vessel development and sprouting angiogenesis (Fig. S3A). Thus, we proposed E02-tip-CXCR4 as the pivotal angiogenic cells in tumors. Notably, tip cells also showed a pronounced collagen formation activity (Fig. 2A and C). Several aforementioned vein or artery subsets, E09, E10, and E16, with the high expression of collagen-associated genes, also demonstrated a high angiogenesis score. By contrast, those subsets with low expression of collagens, such as E06, E07, E08, and E15, featured inferior angiogenesis potential (Fig. 2B and C). Strikingly, for all ECs, we found a clear positive correlation between angiogenesis and collagen formation score (Fig. 2E). Taking into account the minimal overlap of these two gene sets (Fig. S3B), we concluded that these two processes might be coupled during the EC state transition. Accordingly, therapeutics targeting collagen formation might be explored as alternative anti-angiogenic strategies.

For the leukocyte-endothelial adhesion, veins were ranked with the top activity (Fig. 2D). Within veins, E06-veins-SELE cells exhibited stronger leukocyte-endothelial adhesion activity than others, consistent with the high expression of *SELE, ICAM1*, and *VCAM1* in E06 (Fig. 2F). Of particular note was that E06 harbored the lowest collagen formation and angiogenesis score within all veins (Fig. 2B and C). In addition, pathways including inflammatory response and regulation of T cell activation were enriched in E06 compared to other vein subsets (Fig. S3C). Leveraging the TCGA RNA-seq datasets, we found that the E06 signature was positively correlated with T cell or CD8 T cell signatures (Figs 2G and S3D; Supplementary Methods). In scRNA-seq data, tumor samples with higher E06 frequencies exhibited abundant T cells but a lower proportion of epithelial or malignant cells (Fig. 2H). As a contrast, samples with higher E02-tip-CXCR4 frequencies showed a lower proportion of T cells but higher epithelial or malignant cells (Fig. 2I). Given these observations, we deduced E06-veins-SELE as the inflammatory response-associated ECs, linking to T cell infiltration in tumors.

**Figure 3.**
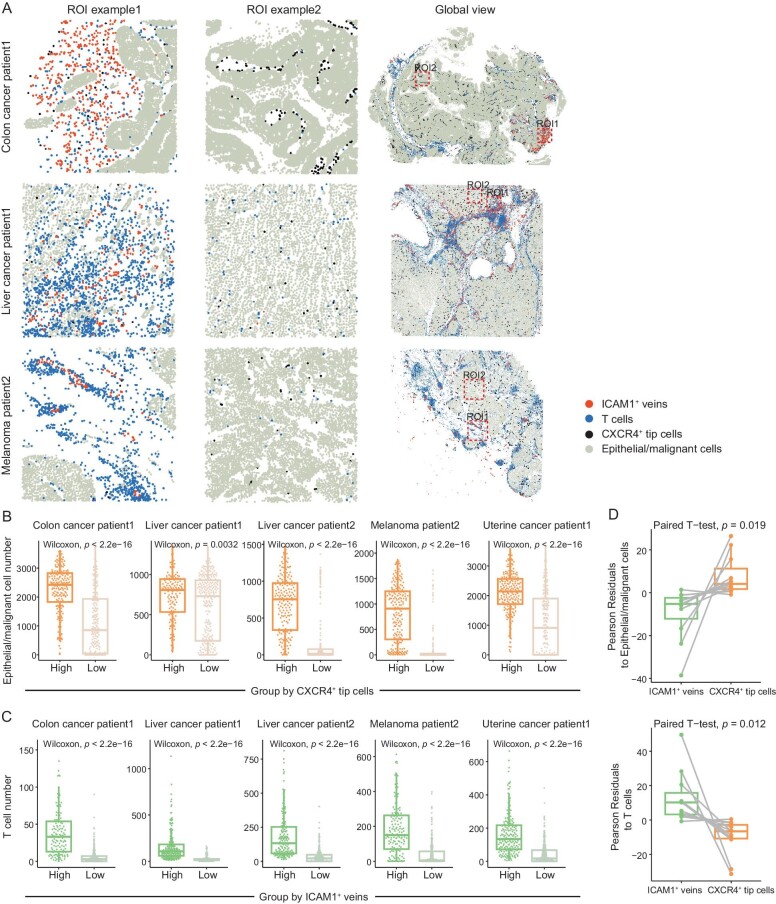
Spatial distribution of *ICAM1*^+^ veins and *CXCR4*^+^ tip cells. (A) Scatter spatial plots showing the distribution patterns of *ICAM1*^+^ veins and *CXCR4*^+^ tip cells in both local and global views in patient samples from colon cancer, liver cancer and melanoma (Supplementary Methods). All local regions covered 1000 $\times$ 1000 unit area. (B and C) Boxplots comparing the numbers of epithelial/malignant cells (B) and T cells (C) between different region groups. Two-sided unpaired Wilcoxon test. (D) Boxplots comparing the Pearson residuals of *ICAM1*^+^ veins and *CXCR4*^+^ tip cells to epithelial/malignant cells (left) and T cells (right) (Supplementary Methods). Paired t-test.

Focusing on E02-tip-CXCR4 and E06-veins-SELE, we applied Cytosig to dissect their potential regulatory cytokine signaling (Supplementary  Methods) [38]. While strong TGFb and CXCL12 activities were observed in E02, these cells appeared not to be mediated by inflammatory-related interleukins and interferons (Fig. 2J). By contrast, the activities of these inflammatory factors were prominent in E06 (Fig. 2K). We then examined the spatial distribution of E02-tip-CXCR4 and E06-veins-SELE using public MERFISH-based spatial data [39]. *CXCR4*^+^ tip cells and *ICAM1*^+^ veins were identified, resembling the transcriptional features of E02 and E06, respectively (Fig. S3E; Supplementary Methods). *CXCR4*^+^ tip cells dispersed in the tumor core and typically co-localized with epithelial or malignant cells. Instead, *ICAM1*^+^ veins tended to be located near T cells spatially (Figs 3A and S4A). To quantify their spatial distribution pattern, we randomly sampled local regions from each MERFISH data, categorizing these regions based on the number of *CXCR4*^+^ tip cells and *ICAM1*^+^ veins. Compared to regions with fewer *CXCR4*^+^ tip cells, those with higher numbers featured a high abundance of epithelial or malignant cells (Figs 3B and S4B). By contrast, T cells were enriched in regions with abundant *ICAM1*^+^ veins (Figs 3C and S4B). Consistently, the whole-slice statistical analysis confirmed the enrichment of T cells within the spatial neighborhood of *ICAM1*^+^ veins, and the enrichment of epithelial or malignant cells within that of *CXCR4*^+^ tip cells (Fig. 3D).

In summary, various EC subsets exhibited divergent functional preferences, and E02-tip-CXCR4 and E06-veins-SELE, positioned in different spatial niches in tumors, may represent two poles of the angiogenic and proinflammatory phenotype of TECs.

### The cross-organ TEC heterogeneity among various cancers

**Figure 4.**
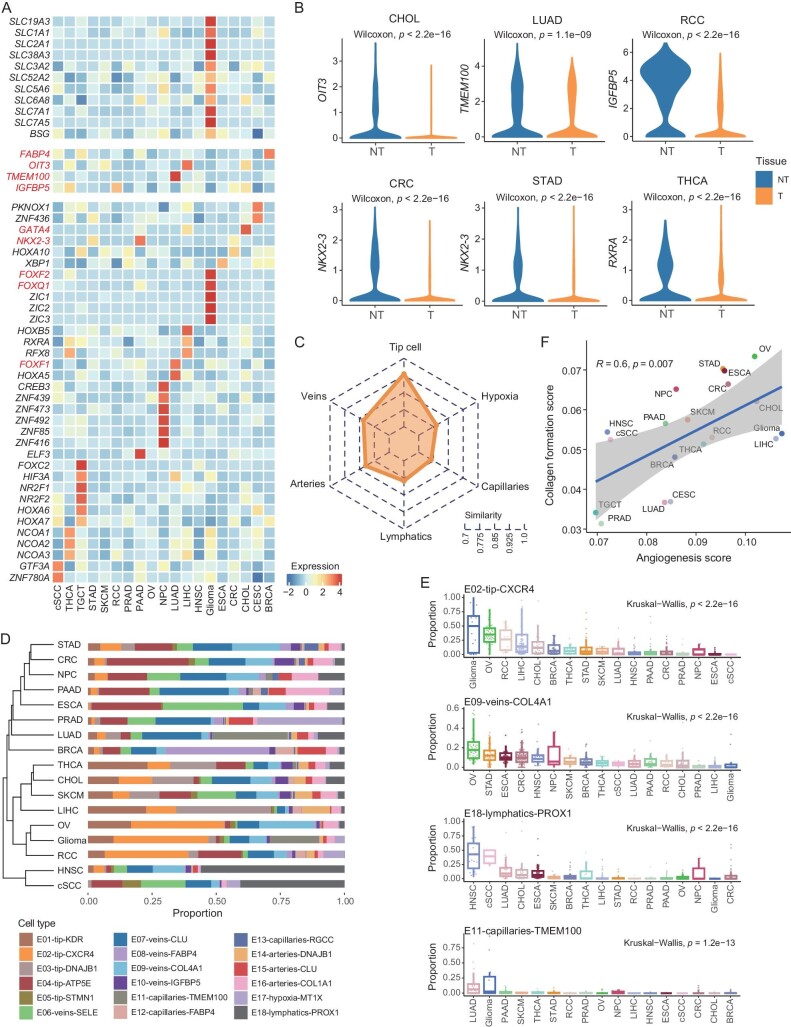
Heterogeneity of TECs across cancer types. (A) Heatmap showing the cancer-type specific gene expression patterns in all analyzed cancer types. Color indicates the Z score scaled gene expression levels. (B) Boxplots comparing the expression of cancer-type specific genes in tumor and adjacent non-tumor tissues. Two-sided unpaired Wilcoxon test. (C) Radar chart showing the transcriptome similarity of each major compartment of ECs among all analyzed cancer types (Supplementary Methods). (D) Unsupervised hierarchical clustering of cancer types based on the cellular composition of all EC subsets in each cancer type. The average proportion of each EC subset is shown. (E) Boxplots showing the varied proportion of EC subsets among cancer types. Kruskal–Wallis test. (F) The positive correlation between angiogenesis score and collagen formation score in each cancer type. Pearson correlation test.

We then investigated whether host organ intrinsic properties of ECs were conserved in tumors. Indeed, multiple organ-specific genes were distinctively harbored by corresponding cancer types (Fig. 4A). Particularly, ECs from glioma exclusively expressed a series of transmembrane transport-associated genes, including *SLC2A1, BSG, SLC7A1*, and *SLC7A5*, suggesting that ECs in brain tumors were still involved in retaining the brain-blood barrier. Other examples of organ-specific gene expression characteristics in tumors included *FABP4* in BRCA, *OIT3* in CHOL and LIHC, and *TMEM100* in LUAD [20,40]. We subsequently obtained pathways enriched in each cancer type based on their top-upregulated genes (Table S3; Supplementary Methods), observing substantial differences between cancers. Consistently, glioma exhibited pronounced enrichment in pathways associated with the maintenance of the blood-brain barrier (Fig. S4C). We also examined the expression pattern of previously reported organ-specific transcription factors (TFs), detecting that their high expression was confined in tumors derived from corresponding organs. For instance, *FOXQ1* and *FOXF2* were highly expressed in glioma, *FOXF1* in LUAD, and *GATA4* in CHOL and LIHC, all consistent with previous studies [25,41–43]. Our large-scale atlas enabled the identification of other cancer type-specific TFs (Fig. 4A). *NR2F1* and *NR2F2*, associated with testis developmental defects [44], were highly expressed in TECs of TCGT. The neural development-associated TFs, *ZIC1, ZIC2*, and *ZIC3* [45], were specifically expressed in glioma. Intriguingly, *NKX2-3*, highly expressed in the ECs of mouse intestine and colon [25], exhibited preferred expression in CRC, STAD, and PAAD. Notably, we noticed a higher expression level of *NKX2-3* in the non-tumor tissues than in tumors for all three cancers. Indeed, organ-specific genes broadly experienced decreased expression from the non-tumor to the corresponding tumor tissues of the same organ (Figs 4B and S4D).

We next quantified the cancer type-heterogeneity of TECs at the major compartment level (Fig. 4C; Supplementary Methods). Capillaries exhibited the largest transcriptome differences across cancers, aligning with their adaptable phenotype to fit specific tissues [25]. Tip cells displayed similar expression profiles across cancer types. Finally, we interrogated differences in the sub-population structure among analyzed cancers (Fig. 4D; Supplementary  Methods). While dramatic variations were observed across cancers, specific patterns still emerged. For example, two subsets with a high expression of *FABP4*, E08-veins-FABP4, and E12-capillaries-FABP4, were enriched in BRCA (Fig. S4E). HNSC and cSCC were characterized by abundant E18-lymphatics-PROX1 (Fig. 4E), which might be potentially explained by the intrinsic rich network of lymph vessels in the head and neck, as well as skin [46]. OV and glioma were clustered together as they both featured a high angiogenesis score. However, OV demonstrated a higher collagen formation score than glioma, and correspondingly, had a uniquely heightened abundance of E09-veins-COL4A1 (Fig. 4E and F). Similar to the cell-level analysis, angiogenesis and collagen formation scores in all analyzed cancers were also positively correlated (Fig. 4F). PRAD featured low angiogenesis and collagen formation scores, and E06-veins-SELE and E07-veins-CLU subsets were abundant in PRAD (Fig. S4E). This might partly explain the poor clinical outcomes of bevacizumab in castration-resistant prostate cancers [47]. Taken together, we depicted the divergence of transcriptional phenotypes and subset proportions in ECs from different cancers and provided evidence that therapeutics targeting angiogenesis should consider such cancer-type heterogeneity.

### Systematic reprogramming of endothelial cells in tumor tissues

**Figure 5.**
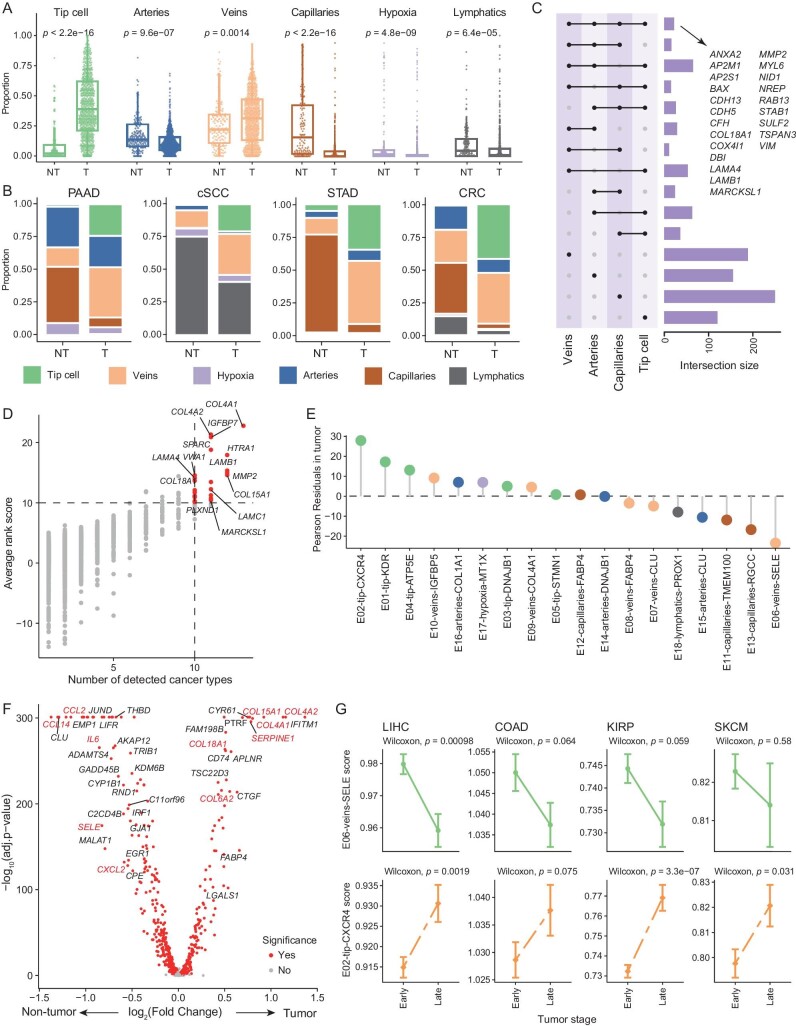
The characteristics and alterations of TECs. (A) Boxplots comparing the proportion of each major compartment of ECs between tumor and adjacent non-tumor tissues. Two-sided unpaired Wilcoxon test. (B) Paired bar plots showing the composition of major compartments in tumor and adjacent non-tumor tissues of representative cancer types. (C) Upset plot displays the intersection size of upregulated genes for four major compartments of blood vascular ECs within tumors. Mode of ‘distinct’ was used. (D) Assessment and selection of the co-upregulated genes in TECs across different cancer types. The x-axis shows the number of cancer types in which the gene was detected as upregulated, and the y-axis shows the average rank score for a gene in all analyzed cancer types. (E) Lollipop plot showing the Pearson Residuals in tumor tissue for all EC subsets (Supplementary Methods). (F) Volcano plot showing differentially expressed genes for venous ECs from the tumor and adjacent non-tumor tissues. Red dots denote genes with adjusted *P*-value < 0.05, two-sided unpaired Wilcoxon test. (G) The variation trends of the E02-tip-CXCR4 and E06-veins-SELE scores between different stages of various cancer types in the TCGA dataset. Data presented as mean ± s.e.m. Two-sided unpaired Wilcoxon test.

The above observations indicated that despite cancer-type heterogeneity, characteristics of ECs were reshaped during cancer development, with certain organ specificities depressed. We next investigated alterations of ECs from adjacent non-tumor to tumors. First, arteries, capillaries, and lymphatics all exhibited a dramatically decreased abundance from non-tumor to tumors (Fig. 5A). The largest magnitude was observed for capillaries, with only limited presence detected in most cancers except LUAD (Figs 5B and S5A). In contrast, tip cells experienced an elevated abundance from non-tumor to tumors (Fig. 5A and B). A previous study has found that <10% of TECs belong to tip cells in human lung tumors, suggesting that targeting only tip cells might not be adequate to block tumor angiogenesis [20]. In our atlas, although tip cells were relatively rare in certain cancers including LUAD, HNSC, and PRAD, they constituted >50% of all TECs in cancers like glioma, LIHC, OV, and RCC (Fig. S5A). In particular, tip cells accounted for nearly 90% of TECs in glioma, which could be attributed to the finding that angiogenesis is critical for the growth and invasiveness of brain tumors [7,18,48].

Our aforementioned analyses demonstrated certain transcriptional characteristics similar across EC major compartments. Therefore, we asked whether consistent changes in gene expressions existed across these major compartments from non-tumor to tumors. Focusing on four compartments of blood vascular ECs, we obtained their respective top-upregulated genes in tumors compared to non-tumor tissues (Table S4; Supplementary Methods). While many genes were upregulated in tumors only for one specific compartment, 22 upregulated genes were identified in all four compartments (Fig. 5C). These genes were enriched in pathways associated with laminin interactions and blood vessel development (Fig. S5B). In addition, the heightened collagen formation in tumors was conserved for all four compartments (Fig. S5C). We next examined whether those 22 genes were conserved across cancers. We first performed differential expression analyses of ECs between non-tumor and tumor tissues, for each cancer type separately. Likewise, we retained top-upregulated genes in tumors for each analysis and compared their overlap (Fig. S5D; Table S5). Notably, *COL4A1* exhibited the highest mean rank scores across cancers, with increased expression observed in almost all cancers. We next screened cancer type-conserved TEC-specific genes and intersected them with the 22 genes. (Fig. 5D). Only seven genes, including *COL18A1, LAMA4, LAMB1, MARCKSL1, MMP2, NID1*, and *RAB13*, were retained, constituting the characteristic gene set of TECs. The mean expression of this gene set achieved an area under the curve (AUC) score of 0.81 when discriminating TECs from NECs (Fig. S5E), confirming the reliable tumor-specificity of this gene set.

Next, we investigated the deviation of ECs in tumors from non-tumor tissues with our fine-grained subsets. Checking the tissue distribution of each subset revealed multiple tumor-enriched subsets (Supplementary Methods), including all tip cell subsets, E12-capillaries-FABP4, and E16-arteries-COL1A1. For veins, we found that E06, E07, and E08, were enriched in non-tumor tissues, whereas other vein subsets, E09 and E10, preferred to distribute in tumors (Fig. 5E). Consistently, a series of chemokines, such as *CCL2, CCL14*, and *CXCL2*, as well as selectins were highly expressed in non-tumor–derived veins, whereas tumor-derived ones showed increased expression of genes related to collagen formation and ECM remodeling (Fig. 5F; Table S6). Among all subsets, E02-tip-CXCR4 and E06-veins-SELE exhibited the highest degree of enrichment in tumors and non-tumor tissues, respectively (Fig. 5E). We then explored their potential evolutionary process during cancer progression. Using TCGA patients from different clinical stages, we could trace the abundance change of these two subsets as cancers evolve from early to late stage (Supplementary Methods). In most cancers, the proinflammatory E06-veins-SELE demonstrated a decreased abundance from early to late stage; by contrast, E02-tip-CXCR4 showed increased enrichment (Figs 5G and S5F). In summary, our observations revealed consistent impacts of cancer development on tumor vasculatures across cancers, and importantly, malignancy might continually induce the polarization of endothelium to pro-tumoral roles.

### Impact of tumor vascular structure on clinical outcomes in cancer patients

**Figure 6.**
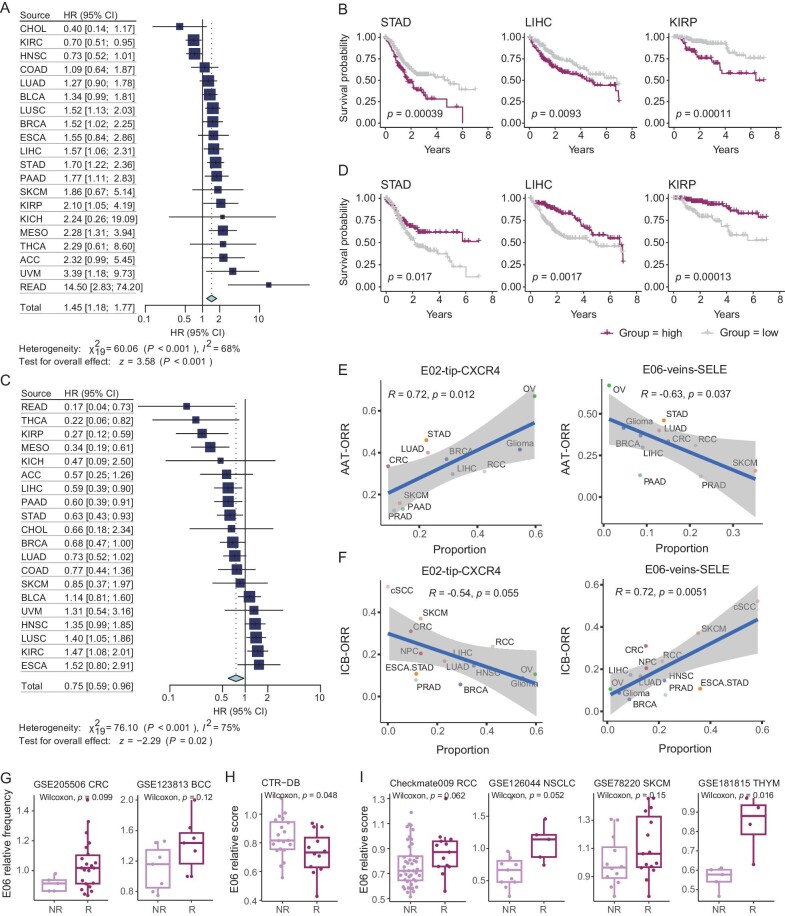
The relationship between the population structure of TEC and clinical outcomes. (A and C) Forest plots showing the effects of E02-tip-CXCR4 cells (A) and the relative enrichment of E06-veins-SELE cells (C) on overall patient survival of each cancer type and pan-cancer level. The hazard ratios are calculated using Cox regression models with the age, gender, and stage corrected. *P* values are adjusted by Benjamini–Hochberg. (B and D) Kaplan–Meier plots showing the association of the signature score of E02-tip-CXCR4 cells (B) and the relative enrichment of E06-veins-SELE cells (D) in tumors with prognosis. +, censored observations; log-rank test. (E) Scatterplot showing the correlation of the proportion of E02-tip-CXCR4 cells (left) or E06-veins-SELE cells (right) with the ORR of the anti-angiogenic therapy (AAT) in various cancer types. Pearson correlation test. (F) Scatterplot showing the correlation of the proportion of E02-tip-CXCR4 cells (left) or E06-veins-SELE cells (right) with the ORR of the immune checkpoint blockade (ICB) in various cancer types. Pearson correlation test. (G) Boxplots comparing the E06-veins-SELE relative frequencies between NRs and Rs in ICB-related scRNA-seq datasets. Two-sided unpaired Wilcoxon test. (H and I) Boxplots comparing the E06-veins-SELE relative scores between non-responders (NRs) and responders (Rs) in AAT (H) and ICB (I) related datasets. Two-sided unpaired Wilcoxon test.

We then interrogated the clinical association of the two poles of angiogenic and proinflammatory phenotypes of ECs, E02-tip-CXCR4 and E06-veins-SELE. Using TCGA datasets, we found that the high expression of the E02 signature genes was linked to poor prognosis in most cancers, and at the pan-cancer level (Figs 6A and B, and S6A; Supplementary Methods). By contrast, patients from multiple cancers with high E06 signatures tended to exhibit favorable survival, albeit with cancer-type heterogeneity (Fig. S6B). Notably, the relative ratio of E06 signature to E02 signature exhibited a clear association with patient survival at the pan-cancer level (Fig. 6C), and a higher relative ratio of E06 typically indicated better clinical outcomes (Figs 6D and S6C). We also employed a deep learning-based model for deconvolution and cell composition analyses [49], confirming that a high relative E06 signature indicated a favorable prognosis among cancer patients (Fig. S6D and E; Supplementary Methods).

We next examined the associations between these two subsets with different cancer therapeutics. Considering the representative angiogenic phenotype of E02, we wondered whether it was correlated with the AAT treatment efficacy. To address this, we collected the objective response rate (ORR) from AAT clinical trials, with the ORR of 11 cancer types obtained (Supplementary Methods) [50–60]. Notably, a strong correlation was observed between the E02 proportion with the ORR for these cancers (*R* = 0.72, *p* = 0.012; Fig. 6E), underscoring that those cancer types with their endothelium compartment dominated by E02 tended to benefit from AAT. Turning to E06, although this cell subset also exhibited a high activity of the VEGFA signaling (Fig. 2K), we observed a negative correlation between E06 abundance and the ORR of AAT (*R* = −0.63, *p* = 0.037; Fig. 6E). Given the close connection of E06 with the infiltration of T cells into tumors, we suspected that such angiogenesis-targeting strategies might collaterally inhibit these proinflammatory ECs. Thus, for those cancer patients with abundant E06 cells, AAT might incur worse clinical outcomes.

The function of E06 to promote T-cell infiltration motivated us to ask whether the TEC composition could be linked to the treatment efficacy of ICB. We then correlated the proportion of E02 and E06 cells with the ORR of ICB in different cancer types [61]. In contrast to the results of AAT, E06 exhibited a strong positive correlation with the ORR of ICB (*R* = 0.72, *p* = 0.0051), while a negative correlation was observed for E02 (*R* = −0.54, *p* = 0.055; Fig. 6F), demonstrating a previously unknown connection between the TEC population structure with the clinical outcome of ICB. Importantly, we did not observe significant clinical associations at the compartment level of tip cells and veins (Fig. S6F and G). Using scRNA-seq data from previous studies on ICB therapy [62,63], we noted a higher E06 relative frequency in responders than non-responders (Fig. 6G; Supplementary Methods). To further consolidate these findings, we collected bulk RNA-seq datasets of ICB or AAT with response information (Supplementary Methods) [64–68]. Consistently, a higher relative E06 signature was found in non-responders of AAT, while for ICB, the opposite tendency was observed (Fig. 6H and I).

In summary, the heterogeneous vascular population structure of different cancers can affect their response to distinct treatment options. In particular, the balance between E02 and E06 could serve as a valuable cellular biomarker to identify specific therapeutic options for cancer patients.

## DISCUSSION

We established a large-scale pan-cancer EC atlas at single-cell resolution, entailing unique opportunities to identify the fine-grained TEC subsets. Tip cells were reliably discerned across cancers, with a strong transcriptional similarity. We highlighted one tip cell subset, E02-tip-CXCR4, which harbored the highest angiogenesis potential among all subsets. Indeed, CXCR4, as the chemokine receptor of CXCL12, has been reported to provide chemotaxis signals to direct the growth of vessels and enhance neovascularization [69–71]. In addition, the CXCL12-CXCR4 axis has also been associated with the egression of T cells in tumors [24], thus the overall impact of targeting CXCR4 on endothelial and T cells needs to be further explored. For veins, we did not distinguish one cluster of high endothelial venule cells (Fig. S2C), which might be due to a lack of lymph node samples [72,73]. Although TECs have been mostly linked to tumor growth and metastasis, we found a proinflammatory subset, E06-veins-SELE, exhibiting unique spatial distribution patterns within tumors and closely correlated with T-cell infiltration. We thus speculate that E06 might represent a crossroad of the AAT and ICB therapeutics, providing clues to overcome the tumor vasculature-associated bottlenecks in T cell–based immunotherapies. However, while we unbiasedly detected a wide spectrum of fine-grained subsets, further research is necessary to determine whether all of them possess distinct biological significance and conceptions. Finally, excluding all technical biases for the integration of public datasets is challenging. Although we adopted stringent criteria for dataset inclusion, potential unknown technical factors, such as imbalanced sample sizes and the variability inherent in current sequencing technologies to capture rare cell types, still might exist among datasets.

From a pan-cancer perspective, we unraveled systematic phenotypic alterations of ECs during tumor progression. ECs typically displayed intrinsic heterogeneity among various organs; in tumors, we found that organ-specificity was conserved to a certain extent. However, most cancers exhibited congruent state transitions, including the enrichment of tip cells in tumors and the dramatic reprogramming of the vasculature. For most cancer types, we observed the depletion of E06-veins-SELE in tumors, and this subset further demonstrated a decreased abundance along with the tumor development. In contrast, an increasing prevalence of the pro-tumor E02-tip-CXCR4 manifested at the pan-cancer level. Interestingly, in PAAD, both E02 and E06 were enriched in late-stage tumors (Fig. S5F). In a previous study of a PAAD mouse model, more ECs that express leukocyte adhesion molecules were identified in late-stage than early-stage [74]. This highlighted the heterogeneity across different cancers. It is worth noting that, despite the subtle changes observed in the scores of E02 and E06 with cancer progression, widespread differences in other clinical characteristics among patients hindered us from ascertaining whether patients at different stages would respond differently to AAT or ICB. In addition, accumulating efforts have been made to screen the characteristics of TECs for expanding the targets of endothelium-based therapeutic strategies. Our pan-cancer collections of ECs are imperative for discovering robust signatures, especially considering the high heterogeneity of TECs among cancer types. Accordingly, we uncovered the characteristic transcriptional signals of TECs which were conserved across different major compartments as well as cancers. These results further demonstrated the consistent impacts of tumor progression on the endothelium at the pan-cancer level.

Despite extensive efforts over the past decades to target angiogenic factors-associated tumor growth and certain clinical success, long-term responses are still lacking and few reliable biomarkers have been established to select appropriate individuals [4,18]. In this study, we ascertained that the proinflammatory and angiogenic statuses of TECs could be linked to the response rate of AATs across different cancer types. Importantly, these two functional statuses were also correlated with the treatment efficacy of ICB. These observations further highlighted the importance of endothelium heterogeneity in determining the response to medical interventions and selecting the optimal treatment option for specific patients. Further studies need to factor in the TEC heterogeneity and the function of TECs to mediate anti-tumor immune responses when developing combination therapies with ICB. In addition, degradation or partial modification of the extracellular matrix has been previously reported to occur under angiogenic stimuli [75]. Given the critical role of collagen as the structural protein in the ECM and the positive correlation we observed between collagen formation and angiogenesis, exploring therapeutics targeting collagen formation should be further pursued as alternative anti-angiogenic strategies.

Collectively, we comprehensively charted the heterogeneity of ECs in tumors from a pan-cancer perspective, which could advance the understanding of EC functions in the TME and provide insights into the pursuit of biomarkers for AAT and ICB as well as the development of treatment strategies targeting the TECs. To facilitate the usage of our data for the research community, an interactive portal (http://pan-endo.cancer-pku.cn/) has been built for visualizing and analyzing our integrative data.

## MATERIALS AND METHODS

Detailed materials and methods are available in the Supplementary data.

## Supplementary Material

nwae231_Supplemental_Files

## Data Availability

The collected public gene expression data can be obtained from the Zenodo with accession number 11473718. Visualization of the scRNA-seq datasets in this study can be found at http://pan-endo.cancer-pku.cn/ (username and password: panE). Detailed information of all public datasets has been provided in Table S1.

## CODE AVAILABILITY

Our analysis code has been uploaded to the GitHub repository (https://github.com/Chaosli98/Pan_Endo).

## References

[1] Binnewies M, Roberts EW, Kersten K et al. Understanding the tumor immune microenvironment (TIME) for effective therapy. Nat Med 2018; 24: 541–50. 10.1038/s41591-018-0014-x 29686425 PMC5998822

[2] Mellman I, Chen DS, Powles T et al. The cancer-immunity cycle: indication, genotype, and immunotype. Immunity 2023; 56: 2188–205. 10.1016/j.immuni.2023.09.011 37820582

[3] Wang D, Liu B, Zhang Z. Accelerating the understanding of cancer biology through the lens of genomics. Cell 2023; 186: 1755–71. 10.1016/j.cell.2023.02.015 37059071

[4] Cleveland AH, Fan Y. Reprogramming endothelial cells to empower cancer immunotherapy. Trends Mol Med 2024; 30: 126–35. 10.1016/j.molmed.2023.11.002 38040601 PMC10922198

[5] Lugano R, Ramachandran M, Dimberg A. Tumor angiogenesis: causes, consequences, challenges and opportunities. Cell Mol Life Sci 2020; 77: 1745–70. 10.1007/s00018-019-03351-7 31690961 PMC7190605

[6] Huinen ZR, Huijbers EJM, van Beijnum JR et al. Anti-angiogenic agents—overcoming tumour endothelial cell anergy and improving immunotherapy outcomes. Nat Rev Clin Oncol 2021; 18: 527–40. 10.1038/s41571-021-00496-y 33833434

[7] Weis SM, Cheresh DA. Tumor angiogenesis: molecular pathways and therapeutic targets. Nat Med 2011; 17: 1359–70. 10.1038/nm.2537 22064426

[8] Jayson GC, Kerbel R, Ellis LM et al. Antiangiogenic therapy in oncology: current status and future directions. Lancet North Am Ed 2016; 388: 518–29. 10.1016/S0140-6736(15)01088-0 26853587

[9] Garcia J, Hurwitz HI, Sandler AB et al. Bevacizumab (Avastin^®^) in cancer treatment: a review of 15 years of clinical experience and future outlook. Cancer Treat Rev 2020; 86: 102017. 10.1016/j.ctrv.2020.102017 32335505

[10] Jain RK . Normalizing tumor vasculature with anti-angiogenic therapy: a new paradigm for combination therapy. Nat Med 2001; 7: 987–9. 10.1038/nm0901-987 11533692

[11] Martin JD, Seano G, Jain RK. Normalizing function of tumor vessels: progress, opportunities, and challenges. Annu Rev Physiol 2019; 81: 505–34. 10.1146/annurev-physiol-020518-114700 30742782 PMC6571025

[12] Schaaf MB, Garg AD, Agostinis P. Defining the role of the tumor vasculature in antitumor immunity and immunotherapy. Cell Death Dis 2018; 9: 115. 10.1038/s41419-017-0061-0 29371595 PMC5833710

[13] Amersfoort J, Eelen G, Carmeliet P. Immunomodulation by endothelial cells—partnering up with the immune system? Nat Rev Immunol 2022; 22: 576–88. 10.1038/s41577-022-00694-4 35288707 PMC8920067

[14] Ruoslahti E . Specialization of tumour vasculature. Nat Rev Cancer 2002; 2: 83–90. 10.1038/nrc724 12635171

[15] Winkler J, Abisoye-Ogunniyan A, Metcalf KJ et al. Concepts of extracellular matrix remodelling in tumour progression and metastasis. Nat Commun 2020; 11: 5120. 10.1038/s41467-020-18794-x 33037194 PMC7547708

[16] Steele MM, Jaiswal A, Delclaux I et al. T cell egress via lymphatic vessels is tuned by antigen encounter and limits tumor control. Nat Immunol 2023; 24: 664–75. 10.1038/s41590-023-01443-y 36849745 PMC10998279

[17] Nagl L, Horvath L, Pircher A et al. Tumor endothelial cells (TECs) as potential immune directors of the tumor microenvironment—new findings and future perspectives. Front Cell Dev Biol 2020; 8: 766. 10.3389/fcell.2020.00766 32974337 PMC7466447

[18] Zeng Q, Mousa M, Nadukkandy AS et al. Understanding tumour endothelial cell heterogeneity and function from single-cell omics. Nat Rev Cancer 2023; 23: 544–64. 10.1038/s41568-023-00591-5 37349410

[19] Maishi N, Annan DA, Kikuchi H et al. Tumor endothelial heterogeneity in cancer progression. Cancers 2019; 11: 1511. 10.3390/cancers11101511 31600937 PMC6826555

[20] Goveia J, Rohlenova K, Taverna F et al. An integrated gene expression landscape profiling approach to identify lung tumor endothelial cell heterogeneity and angiogenic candidates. Cancer Cell 2020; 37: 21–36.e13. 10.1016/j.ccell.2019.12.001 31935371

[21] Carmeliet P, De Smet F, Loges S et al. Branching morphogenesis and antiangiogenesis candidates: tip cells lead the way. Nat Rev Clin Oncol 2009; 6: 315–26. 10.1038/nrclinonc.2009.64 19483738

[22] Tang F, Li J, Qi L et al. A pan-cancer single-cell panorama of human natural killer cells. Cell 2023; 186: 4235–51. 10.1016/j.cell.2023.07.034 37607536

[23] Zheng L, Qin S, Si W et al. Pan-cancer single-cell landscape of tumor-infiltrating T cells. Science 2021; 374: abe6474. 10.1126/science.abe6474 34914499

[24] Heidegger I, Fotakis G, Offermann A et al. Comprehensive characterization of the prostate tumor microenvironment identifies CXCR4/CXCL12 crosstalk as a novel antiangiogenic therapeutic target in prostate cancer. Mol Cancer 2022; 21: 132. 10.1186/s12943-022-01597-7 35717322 PMC9206324

[25] Kalucka J, de Rooij LPMH, Goveia J et al. Single-cell transcriptome atlas of murine endothelial cells. Cell 2020; 180: 764–79. 10.1016/j.cell.2020.01.015 32059779

[26] Schaum N, Karkanias J, Neff NF et al. Single-cell transcriptomics of 20 mouse organs creates a *Tabula Muris*. Nature 2018; 562: 367–72. 10.1038/s41586-018-0590-4 30283141 PMC6642641

[27] Qian J, Olbrecht S, Boeckx B et al. A pan-cancer blueprint of the heterogeneous tumor microenvironment revealed by single-cell profiling. Cell Res 2020; 30: 745–62. 10.1038/s41422-020-0355-0 32561858 PMC7608385

[28] Zhang J, Lu T, Lu S et al. Single-cell analysis of multiple cancer types reveals differences in endothelial cells between tumors and normal tissues. Comput Struct Biotechnol J 2023; 21: 665–76. 10.1016/j.csbj.2022.12.049 36659929 PMC9826920

[29] Schulkens IA, Castricum KCM, Weijers EM et al. Expression, Regulation and Function of Human Metallothioneins in Endothelial Cells. J Vasc Res 2014; 51: 231–8. 10.1159/000365550 25116857

[30] Adams RH, Alitalo K. Molecular regulation of angiogenesis and lymphangiogenesis. Nat Rev Mol Cell Biol 2007; 8: 464–78. 10.1038/nrm2183 17522591

[31] Lisabeth EM, Falivelli G, Pasquale EB. Eph receptor signaling and ephrins. Cold Spring Harb Perspect Biol 2013; 5: a009159. 10.1101/cshperspect.a009159 24003208 PMC3753714

[32] Travisano SI, Oliveira VL, Prados B et al. Coronary arterial development is regulated by a Dll4-Jag1-EphrinB2 signaling cascade. eLife 2019; 8: e49977. 10.7554/eLife.49977 31789590 PMC6917494

[33] Murai KK, Pasquale EB. ‘Eph'ective signaling: forward, reverse and crosstalk. J Cell Sci 2003; 116: 2823–32. 10.1242/jcs.00625 12808016

[34] Hao Q, Vadgama JV, Wang P. CCL2/CCR2 signaling in cancer pathogenesis. Cell Commun Signal 2020; 18: 82. 10.1186/s12964-020-00589-8 32471499 PMC7257158

[35] Shetty S, Weston CJ, Oo YH et al. Common lymphatic endothelial and vascular endothelial receptor-1 mediates the transmigration of regulatory T cells across human hepatic sinusoidal endothelium. J Immunol 2011; 186: 4147–55. 10.4049/jimmunol.1002961 21368224 PMC6016742

[36] Wang L, Fan J, Thompson LF et al. CD73 has distinct roles in nonhematopoietic and hematopoietic cells to promote tumor growth in mice. J Clin Invest 2011; 121: 2371–82. 10.1172/JCI45559 21537079 PMC3104756

[37] Eichmann A, Makinen T, Alitalo K. Neural guidance molecules regulate vascular remodeling and vessel navigation. Genes Dev 2005; 19: 1013–21. 10.1101/gad.1305405 15879551

[38] Jiang P, Zhang Y, Ru B et al. Systematic investigation of cytokine signaling activity at the tissue and single-cell levels. Nat Methods 2021; 18: 1181–91. 10.1038/s41592-021-01274-5 34594031 PMC8493809

[39] Vizgen MERFISH FFPE Human Immuno-oncology Data Set. 2022.

[40] Li Z-W, Ruan B, Yang P-J et al. Oit3, a promising hallmark gene for targeting liver sinusoidal endothelial cells. Sig Transduct Target Ther 2023; 8: 344. 10.1038/s41392-023-01621-2 PMC10495338 37696816

[41] Cai Y, Bolte C, Le T et al. FOXF1 maintains endothelial barrier function and prevents edema after lung injury. Sci Signal 2016; 9: ra40. 10.1126/scisignal.aad1899 27095594

[42] Géraud C, Koch PS, Zierow J et al. GATA4-dependent organ-specific endothelial differentiation controls liver development and embryonic hematopoiesis. J Clin Invest 2017; 127: 1099–114. 10.1172/JCI90086 28218627 PMC5330741

[43] Hupe M, Li MX, Kneitz S et al. Gene expression profiles of brain endothelial cells during embryonic development at bulk and single-cell levels. Sci Signal 2017; 10: eaag2476. 10.1126/scisignal.aag2476 28698213

[44] Qin G, Mallik S, Mitra R et al. MicroRNA and transcription factor co-regulatory networks and subtype classification of seminoma and non-seminoma in testicular germ cell tumors. Sci Rep 2020; 10: 852. 10.1038/s41598-020-57834-w 31965022 PMC6972857

[45] Inoue T, Ota M, Ogawa M et al. *Zic1* and *Zic3* regulate medial forebrain development through expansion of neuronal progenitors. J Neurosci 2007; 27: 5461–73. 10.1523/JNEUROSCI.4046-06.2007 17507568 PMC6672357

[46] Breslin JW, Yang Y, Scallan JP et al. Lymphatic vessel network structure and physiology. Compr Physiol 2018; 9: 207–99. 10.1002/cphy.c180015 30549020 PMC6459625

[47] Kelly WK, Halabi S, Carducci MA et al. A randomized, double-blind, placebo-controlled phase III trial comparing docetaxel, prednisone, and placebo with docetaxel, prednisone, and bevacizumab in men with metastatic castration-resistant prostate cancer (mCRPC): survival results of CALGB 90401. J Clin Oncol 2010; 28: LBA4511. 10.1200/jco.2010.28.18_suppl.lba4511 PMC3383121 22454414

[48] Ricci-Vitiani L, Pallini R, Biffoni M et al. Tumour vascularization via endothelial differentiation of glioblastoma stem-like cells. Nature 2010; 468: 824–8. 10.1038/nature09557 21102434

[49] Menden K, Marouf M, Oller S et al. Deep learning-based cell composition analysis from tissue expression profiles. Sci Adv 2020; 6: eaba2619. 10.1126/sciadv.aba2619 32832661 PMC7439569

[50] Qu CY, Zheng Y, Zhou M et al. Value of bevacizumab in treatment of colorectal cancer: a meta-analysis. World J Gastroenterol 2015; 21: 5072–80. 10.3748/wjg.v21.i16.5072 25945023 PMC4408482

[51] Liu Y, Li HM, Wang R. Effectiveness and safety of adding bevacizumab to platinum-based chemotherapy as first-line treatment for advanced non-small-cell lung cancer: a meta-analysis. Front Med 2021; 8: 616380. 10.3389/fmed.2021.616380 PMC8277997 34277647

[52] Ohtsu A, Shah MA, Van Cutsem E et al. Bevacizumab in combination with chemotherapy as first-line therapy in advanced gastric cancer: a randomized, double-blind, placebo-controlled phase III study. J Clin Oncol 2011; 29: 3968–76. 10.1200/JCO.2011.36.2236 21844504

[53] Qi WX, Fu S, Zhang Q et al. Efficacy and toxicity of anti-VEGF agents in patients with castration-resistant prostate cancer: a meta-analysis of prospective clinical studies. Asian Pac J Cancer Prev 2014; 15: 8177–82. 10.7314/APJCP.2014.15.19.8177 25339002

[54] Kindler HL, Niedzwiecki D, Hollis D et al. A double-blind, placebo-controlled, randomized phase III trial of gemcitabine (G) plus bevacizumab (B) versus gemcitabine plus placebo (P) in patients (pts) with advanced pancreatic cancer (PC): a preliminary analysis of Cancer and Leukemia Group B (CALGB). J Clin Oncol 2007; 25: 4508. 10.1200/jco.2007.25.18_suppl.4508

[55] Han X, Ge P, Liu S et al. Efficacy and safety of bevacizumab in patients with malignant melanoma: a systematic review and PRISMA-compliant meta-analysis of randomized controlled trials and non-comparative clinical studies. Front Pharmacol 2023; 14: 1163805. 10.3389/fphar.2023.1163805 37521468 PMC10374288

[56] Miller K, Wang M, Gralow J et al. Paclitaxel plus bevacizumab versus paclitaxel alone for metastatic breast cancer. N Engl J Med 2007; 357: 2666–76. 10.1056/NEJMoa072113 18160686

[57] Finn RS, Qin S, Ikeda M et al. IMbrave150: updated overall survival (OS) data from a global, randomized, open-label phase III study of atezolizumab (atezo) + bevacizumab (bev) versus sorafenib (sor) in patients (pts) with unresectable hepatocellular carcinoma (HCC). J Clin Oncol 2021; 39: 267. 10.1200/JCO.2021.39.3_suppl.267

[58] Escudier BJ, Bellmunt J, Negrier S et al. Final results of the phase III, randomized, double-blind AVOREN trial of first-line bevacizumab (BEV) + interferon-α2a (IFN) in metastatic renal cell carcinoma (mRCC). J Clin Oncol 2009; 27: 5020. 10.1200/jco.2009.27.15_suppl.5020

[59] Perren TJ, Swart AM, Pfisterer J et al. A phase 3 trial of bevacizumab in ovarian cancer. N Engl J Med 2011; 365: 2484–96. 10.1056/NEJMoa1103799 22204725

[60] Wick W, Gorlia T, Bendszus M et al. Lomustine and bevacizumab in progressive glioblastoma. N Engl J Med 2017; 377: 1954–63. 10.1056/NEJMoa1707358 29141164

[61] Yarchoan M, Albacker LA, Hopkins AC et al. PD-L1 expression and tumor mutational burden are independent biomarkers in most cancers. JCI Insight 2019; 4: e126908. 10.1172/jci.insight.126908 30895946 PMC6482991

[62] Yost KE, Satpathy AT, Wells DK et al. Clonal replacement of tumor-specific T cells following PD-1 blockade. Nat Med 2019; 25: 1251–9. 10.1038/s41591-019-0522-3 31359002 PMC6689255

[63] Li J, Wu C, Hu H et al. Remodeling of the immune and stromal cell compartment by PD-1 blockade in mismatch repair-deficient colorectal cancer. Cancer Cell 2023; 41: 1152–69.e7. 10.1016/j.ccell.2023.04.011 37172580

[64] Cho J-W, Hong MH, Ha S-J et al. Genome-wide identification of differentially methylated promoters and enhancers associated with response to anti-PD-1 therapy in non-small cell lung cancer. Exp Mol Med 2020; 52: 1550–63. 10.1038/s12276-020-00493-8 32879421 PMC8080767

[65] Hugo W, Zaretsky JM, Sun L et al. Genomic and transcriptomic features of response to anti-PD-1 therapy in metastatic melanoma. Cell 2016; 165: 35–44. 10.1016/j.cell.2016.02.065 26997480 PMC4808437

[66] He Y, Ramesh A, Gusev Y et al. Molecular predictors of response to pembrolizumab in thymic carcinoma. Cell Rep Med 2021; 2: 100392. 10.1016/j.xcrm.2021.100392 34622229 PMC8484507

[67] Liu Z, Liu J, Liu X et al. CTR-DB, an omnibus for patient-derived gene expression signatures correlated with cancer drug response. Nucleic Acids Res 2022; 50: D1184–99. 10.1093/nar/gkab860 34570230 PMC8728209

[68] Choueiri TK, Fishman MN, Escudier B et al. Immunomodulatory activity of nivolumab in metastatic renal cell carcinoma. Clin Cancer Res 2016; 22: 5461–71. 10.1158/1078-0432.CCR-15-2839 27169994 PMC5106340

[69] Tachibana K, Hirota S, Iizasa H et al. The chemokine receptor CXCR4 is essential for vascularization of the gastrointestinal tract. Nature 1998; 393: 591–4. 10.1038/31261 9634237

[70] Ara T, Tokoyoda K, Okamoto R et al. The role of CXCL12 in the organ-specific process of artery formation. Blood 2005; 105: 3155–61. 10.1182/blood-2004-07-2563 15626744

[71] Strasser GA, Kaminker JS, Tessier-Lavigne M. Microarray analysis of retinal endothelial tip cells identifies CXCR4 as a mediator of tip cell morphology and branching. Blood 2010; 115: 5102–10. 10.1182/blood-2009-07-230284 20154215

[72] Veerman K, Tardiveau C, Martins F et al. Single-cell analysis reveals heterogeneity of high endothelial venules and different regulation of genes controlling lymphocyte entry to lymph nodes. Cell Rep 2019; 26: 3116–31. 10.1016/j.celrep.2019.02.042 30865898

[73] Moussion C, Girard J-P. Dendritic cells control lymphocyte entry to lymph nodes through high endothelial venules. Nature 2011; 479: 542–6. 10.1038/nature10540 22080953

[74] Schlesinger Y, Yosefov-Levi O, Kolodkin-Gal D et al. Single-cell transcriptomes of pancreatic preinvasive lesions and cancer reveal acinar metaplastic cells’ heterogeneity. Nat Commun 2020; 11: 4516. 10.1038/s41467-020-18207-z 32908137 PMC7481797

[75] Neve A, Cantatore FP, Maruotti N et al. Extracellular matrix modulates angiogenesis in physiological and pathological conditions. Biomed Res Int 2014; 2014: 756078. 10.1155/2014/756078 24949467 PMC4052469

