# Supplementary material for: Pan-cancer integrative analyses dissect the remodeling of endothelial cells in human cancers
Source: Natl Sci Rev. 2024 Jul 11;11(9):nwae231. doi: 10.1093/nsr/nwae231 (PMC11429526; doi:10.1093/nsr/nwae231)
Supplement: nwae231_Supplemental_Files [file nwae231_supplemental_files.zip › Supplementary figures and legend.docx]

**Supplementary figures**


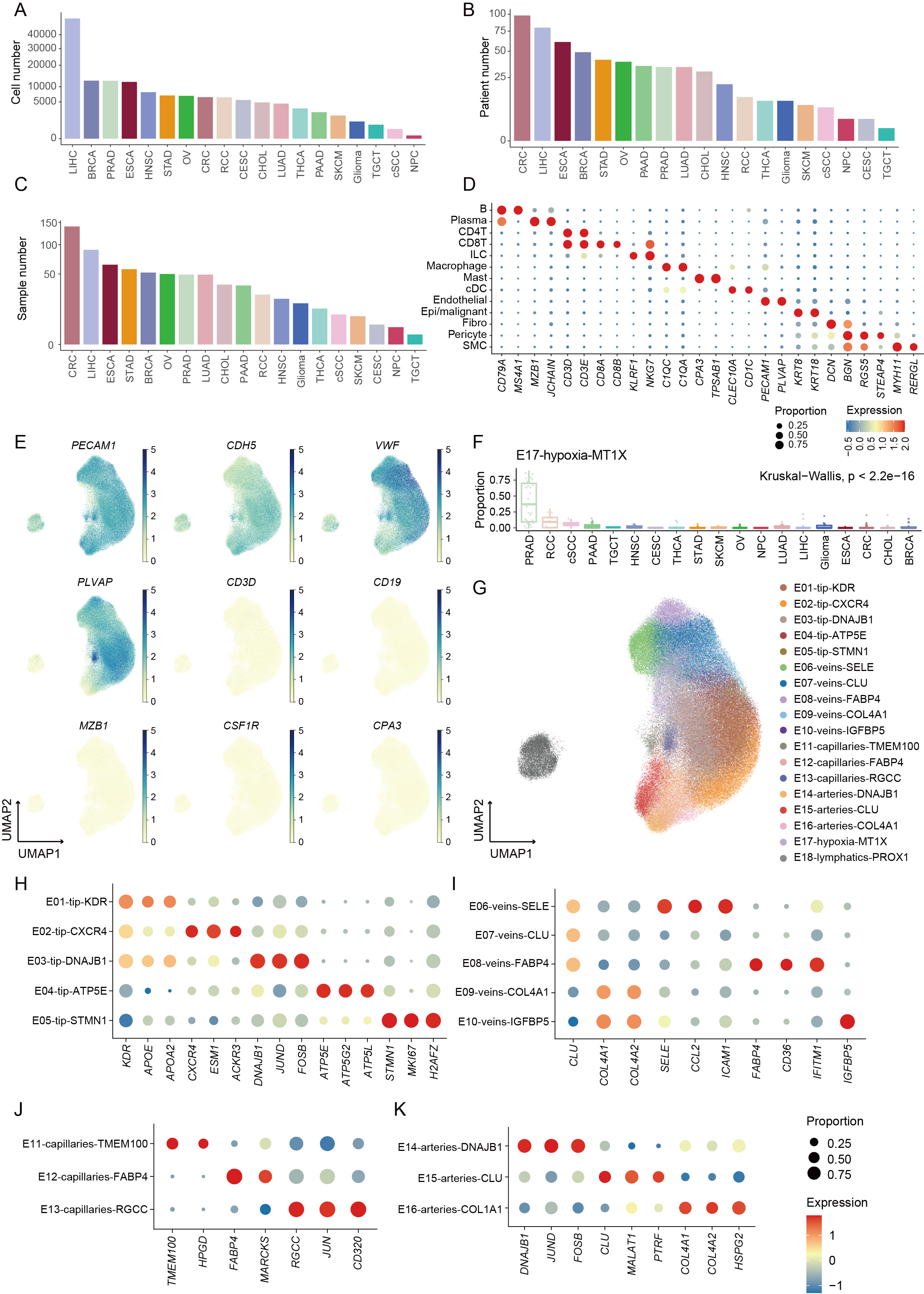


**Figure S1. Basic information of the integrative data**

(A-C) The number of cells (A), patients (B), and samples (C) across cancer types. The y-axis is scaled by a square root transformation.

(D) Bubble heatmap showing the expression patterns for the signature genes of major cell populations. Dot size represents the proportion of expressing cells. Color indicates the Z score scaled gene expression levels.

(E) UMAP visualizations of the marker genes of major cell populations in integrative high-quality ECs.

(F) Boxplot comparing the proportion of E17-hypoxia-MT1X across cancer types. Kruskal-Wallis test.

(G) UMAP visualizations of all fine-grained subsets within all integrative ECs.

(H-K) Bubble heatmaps showing the expression patterns of signature genes of all fine-grained subsets within corresponding major compartments, including tip cells (H), veins (I), capillaries (J), and arteries (K). Dot size represents the proportion of expressing cells. Color indicates the Z score scaled gene expression levels.


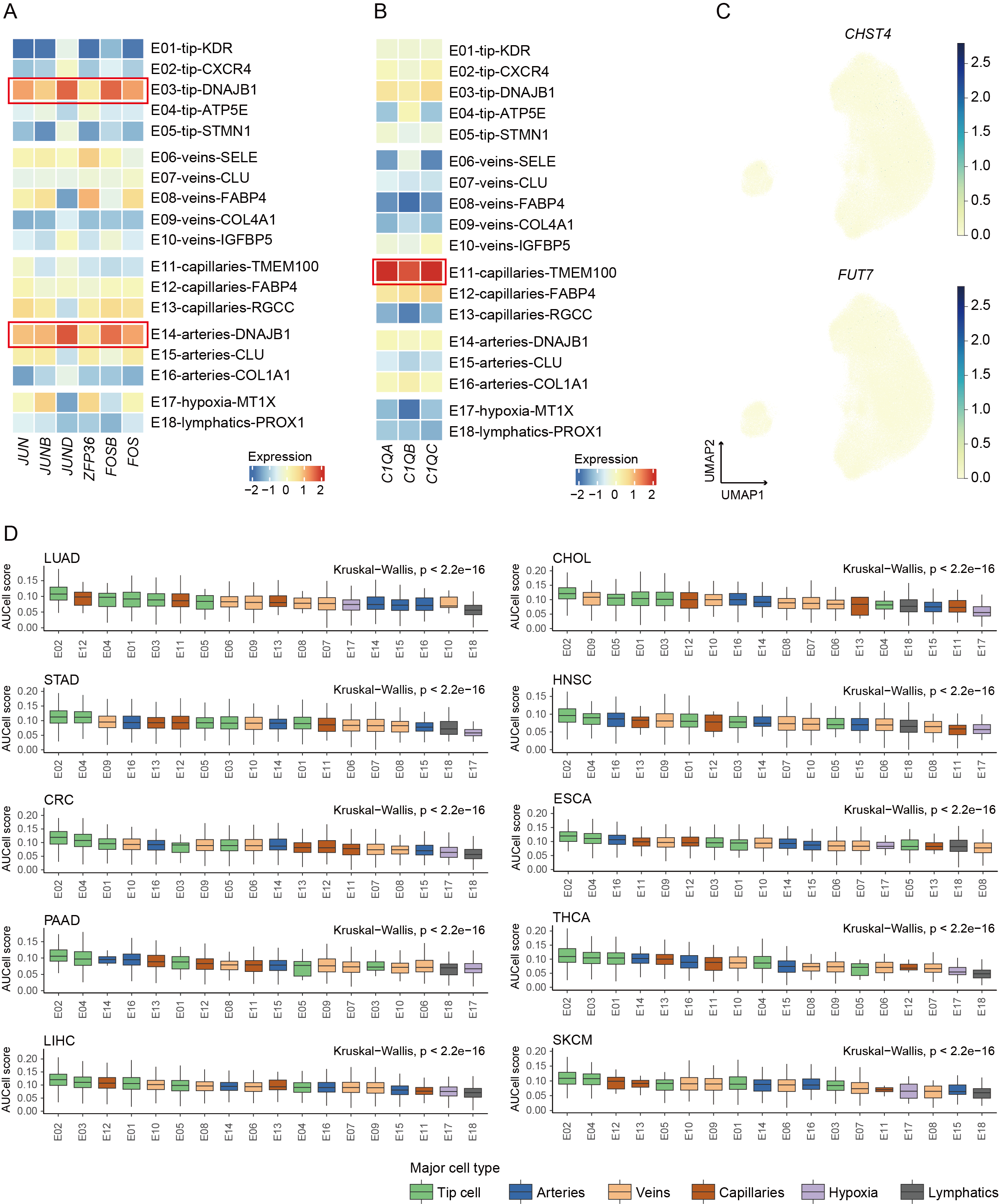


**Figure S2. Characteristics of endothelial cell subsets**

(A-B) Heatmaps showing the expression patterns of stress response-associated genes (A) and complement-relevant genes (B) in EC subsets. Color indicates the Z score scaled gene expression levels.

(C) UMAP visualizations of the marker genes of high endothelial venule cells.

(D) Boxplots comparing the angiogenesis score of all endothelial subsets across cancer types. Kruskal-Wallis test


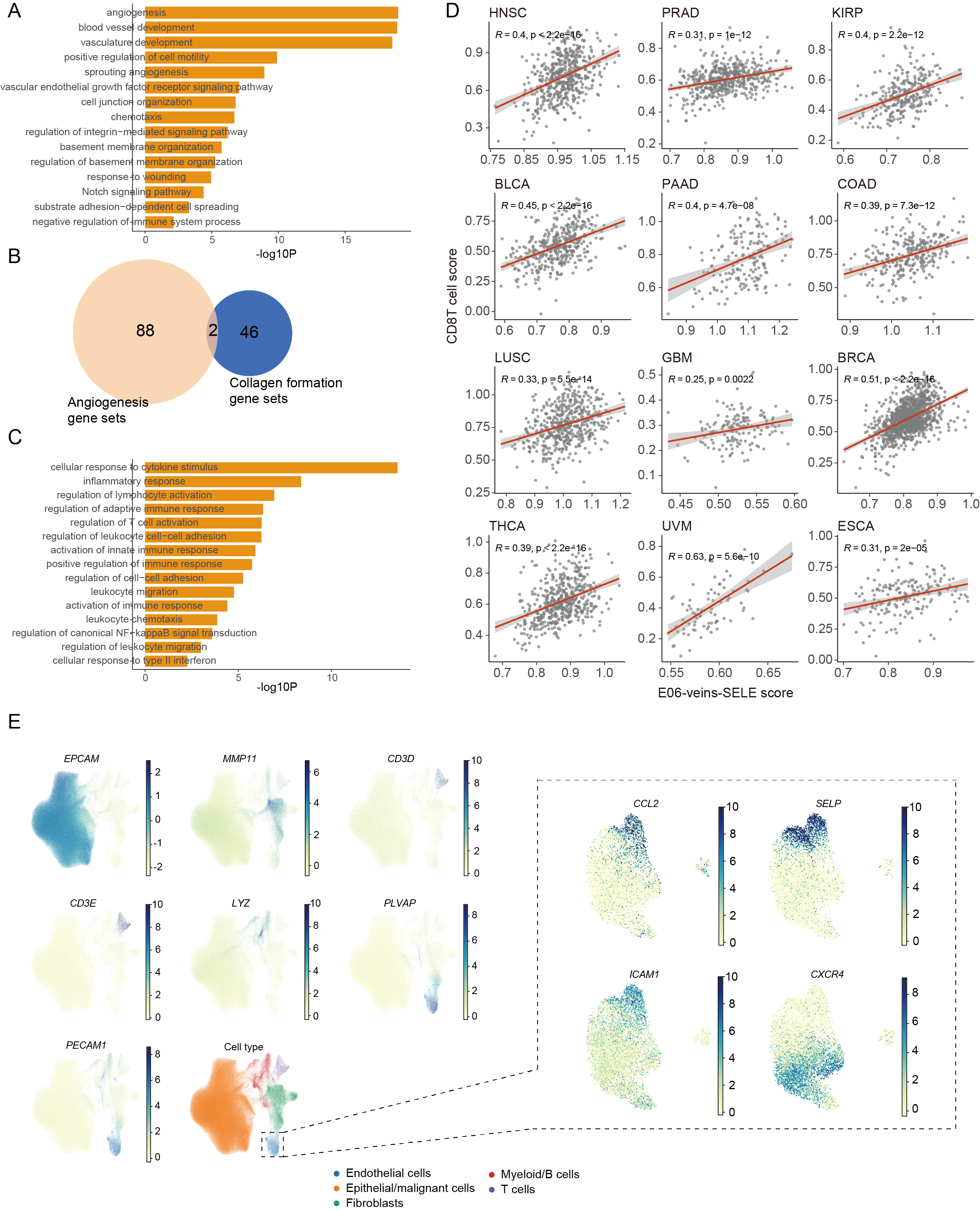


**Figure S3. Functional properties of E02-tip-CXCR4 and E06-veins-SELE cells and processing of the MERFISH data**

(A) Pathways enriched in E02-tip-CXCR4 compared with other tip cell subsets by using Metascape. Hypergeometric test. Benjamini-Hochberg adjusted p value < 0.01.

(B) Venn plot showing the intersection between the gene sets of angiogenesis score and collagen formation score.

(C) Pathways enriched in E06-veins-SELE compared with other venous cell subsets by using Metascape. Hypergeometric test. Benjamini-Hochberg adjusted p value < 0.01.

(D) Scatterplot showing the correlations of E06-veins-SELE with CD8 T cell in each cancer type of TCGA dataset. Each dot represents a tumor sample. Pearson correlation test.

(E) Unsupervised clustering pipeline for the identification of major cell populations, *ICAM1*^+^ veins, and *CXCR4*^+^ tip cells, exemplified by the colon cancer patient (slice1) (Supplementary Methods).


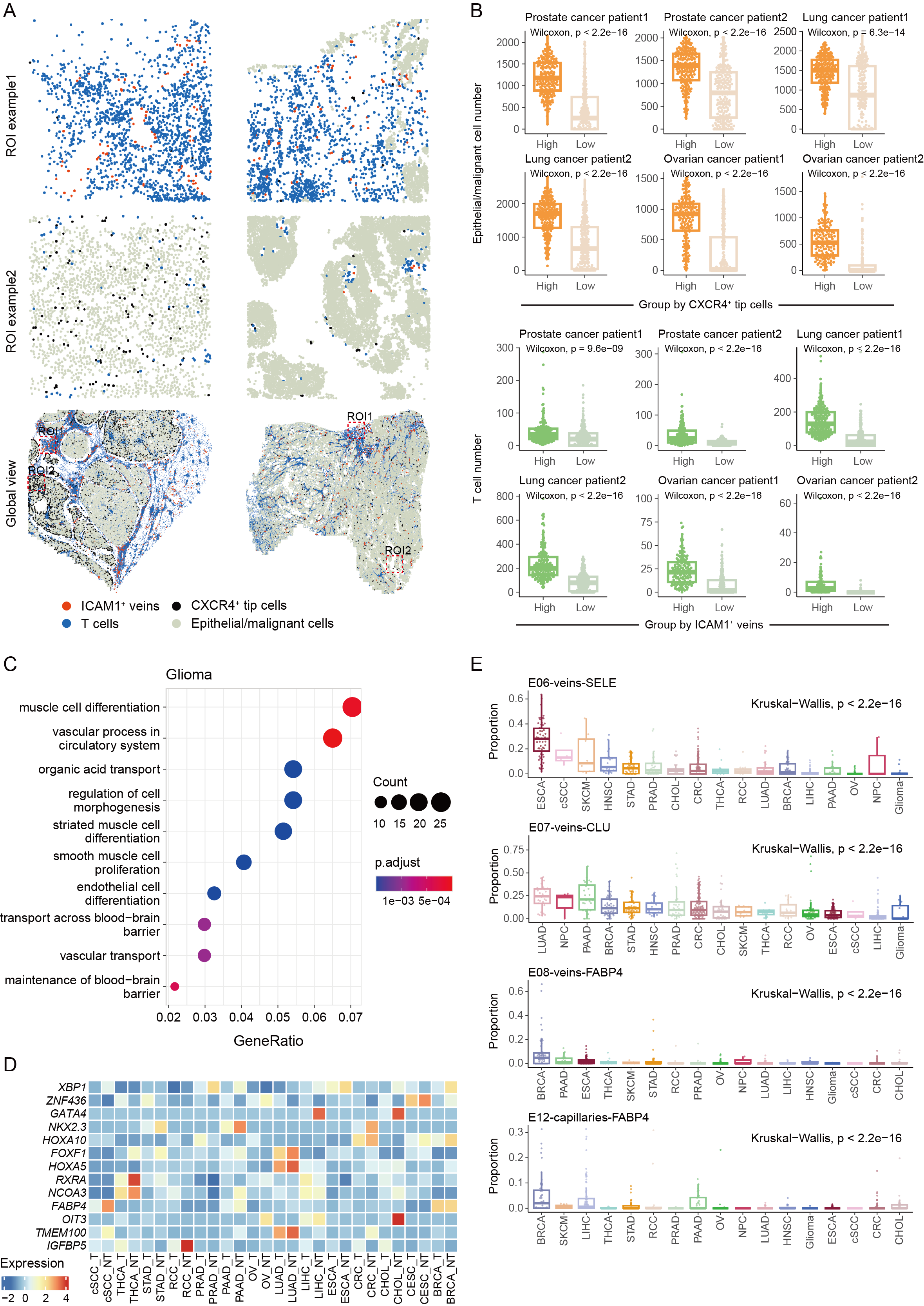


**Figure S4. Spatial distribution of *ICAM1*^+^ veins and *CXCR4*^+^ tip cells and the heterogeneity of TECs**

(A) Scatter spatial plots showing the distribution patterns of *ICAM1*^+^ veins and *CXCR4*^+^ tip cells in both local and global views in liver cancer (slice2) (left) and uterine cancer patient (right) samples.

(B) Boxplots comparing the numbers of epithelial/malignant cells (up) and T cells (bottom) between different region groups. Two-sided unpaired Wilcoxon test.

(C) Dot plot showing the specific Gene Ontology pathways that enriched in the TECs of Glioma. P values are calculated by the Hypergeometric test and adjusted by Benjamini-Hochberg.

(D) Heatmap showing the expression patterns of organ-specific genes in paired tumor and adjacent non-tumor tissues of each cancer type. Color indicates the Z score scaled gene expression levels.

(E) Boxplots showing the varied proportions of EC subsets across cancer types. Kruskal-Wallis test.


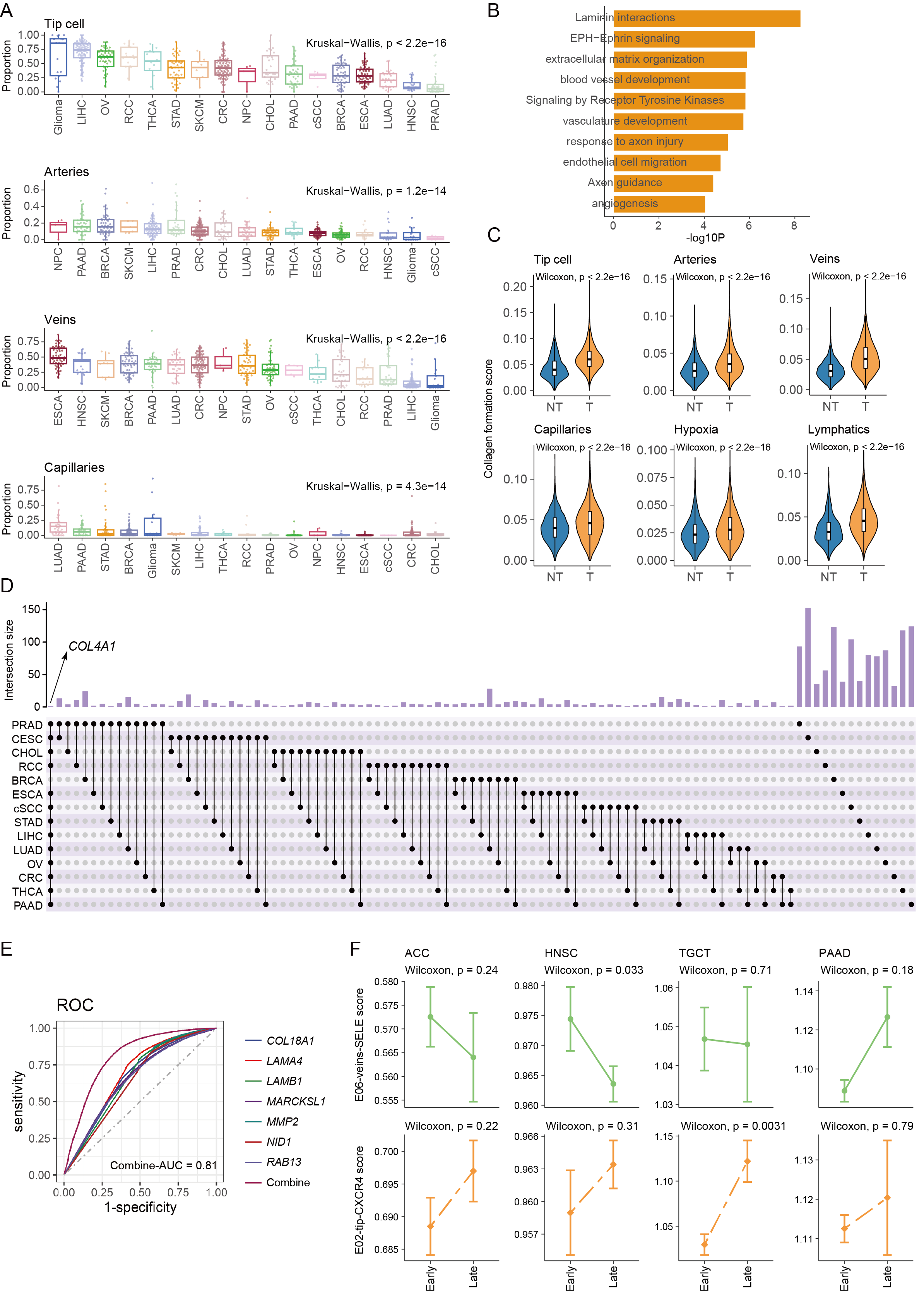


**Figure S5. Pan-cancer characteristics of TECs**

(A) Boxplots showing the varied proportions of major compartments of ECs across cancer types. Kruskal-Wallis test.

(B) Pathways enriched in the co-upregulated genes of all major compartments of ECs by using Metascape. Hypergeometric test. Benjamini-Hochberg adjusted p value < 0.01.

(C) Boxplots comparing the collagen formation scores in tumor and adjacent non-tumor tissues of each major compartment. Two-sided unpaired Wilcoxon test.

(D) Upset plot displays the intersection size of upregulated genes for TECs in each cancer type and their combinations. Mode of “distinct” was used.

(E) Receiver Operator Characteristic (ROC) curves showing the performance of signature gene set in TEC prediction. The dashed line represents the result of random selection.

(F) The variation trends of the E02-tip-CXCR4 and E06-veins-SELE scores between different stages of various cancer types in the TCGA dataset. Data presented as mean ± s.e.m. Two-sided unpaired Wilcoxon test.


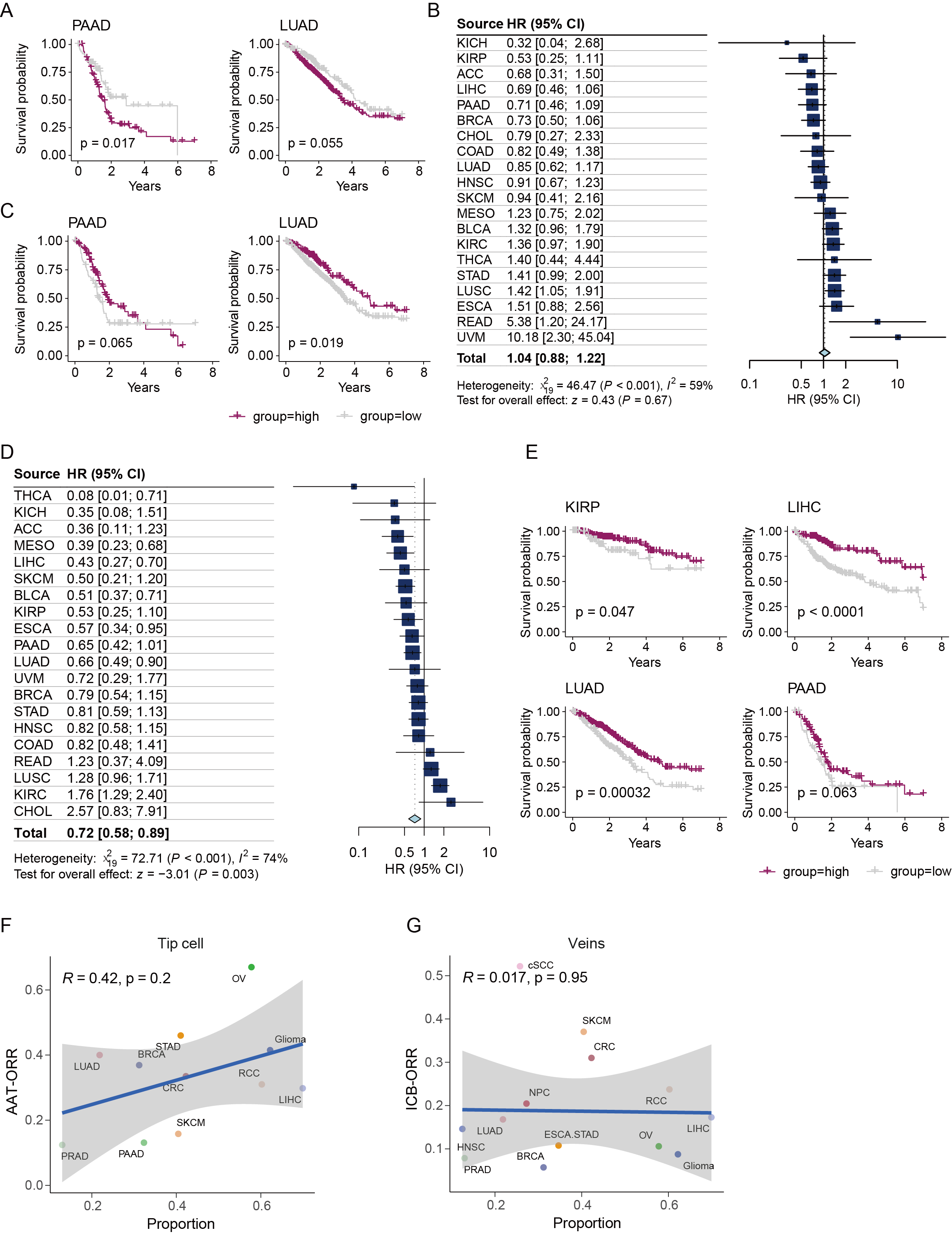


**Figure S6. The relationship between TEC subsets and overall survival of cancer patients**

(A and C) Kaplan-Meier plots showing the association of the signature score of E02-tip-CXCR4 cells (A) and the relative enrichment of E06-veins-SELE cells (C) in tumors with prognosis. +, censored observations; log-rank test.

(B) Forest plots showing the effects of E06-veins-SELE cells on overall patient survival of each cancer type and pan-cancer level. The hazard ratios are calculated using Cox regression models with the age, gender, and stage corrected. P values are adjusted by Benjamini-Hochberg.

(D) Forest plots showing the effects of the relative enrichment of E06-veins-SELE cells on overall patient survival of each cancer type and pan-cancer level based on the deconvolution results. The hazard ratios are calculated using Cox regression models with the age, gender, and stage corrected. P values are adjusted by Benjamini-Hochberg.

(E) Kaplan-Meier plots showing the association of the relative enrichment of E06-veins-SELE cells in tumors with prognosis based on the deconvolution results. +, censored observations; log-rank test.

(F and G) Scatterplots showing the correlation of tip cell proportion with the ORR of AAT (F) and veins proportion with the ORR of ICB (B) in various cancer types. Pearson correlation test.
