# Supplementary material for: Pan-cancer integrative analyses dissect the remodeling of endothelial cells in human cancers
Source: Natl Sci Rev. 2024 Jul 11;11(9):nwae231. doi: 10.1093/nsr/nwae231 (PMC11429526; doi:10.1093/nsr/nwae231)
Supplement: nwae231_Supplemental_Files [file nwae231_supplemental_files.zip › Supplementary figures/sfig6.pdf]

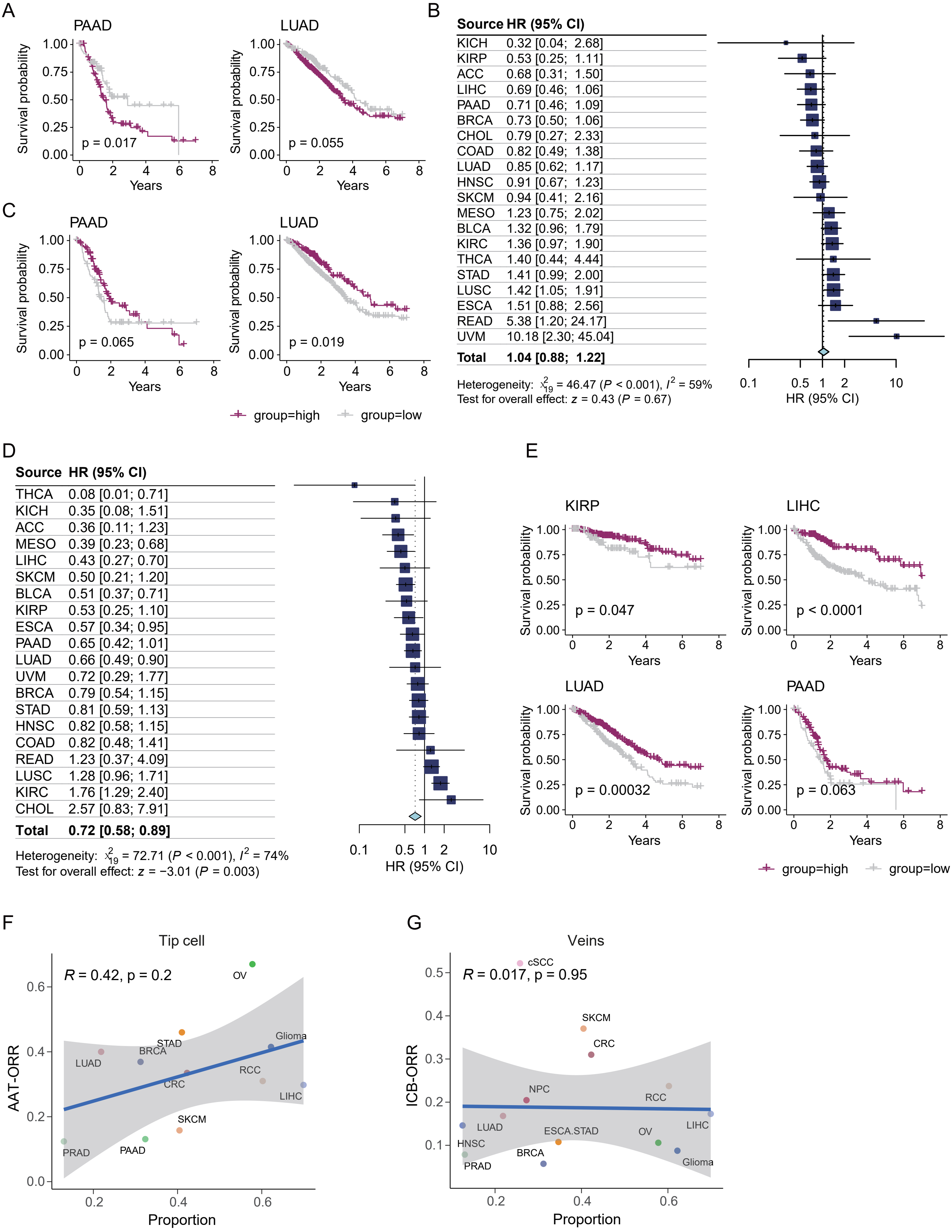

A
PAAD
LUAD
Survival probability
1.00
0.75
0.50
0.25
0.00
p = 0.017
p = 0.055
Years
B
Source HR (95% CI)
KICH 0.32 [0.04; 2.68]
KIRP 0.53 [0.25; 1.11]
ACC 0.68 [0.31; 1.50]
LIHC 0.69 [0.46; 1.06]
PAAD 0.71 [0.46; 1.09]
BRCA 0.73 [0.50; 1.06]
CHOL 0.79 [0.27; 2.33]
COAD 0.82 [0.49; 1.38]
LUAD 0.85 [0.62; 1.17]
HNSC 0.91 [0.67; 1.23]
SKCM 0.94 [0.41; 2.16]
MESO 1.23 [0.75; 2.02]
BLCA 1.32 [0.96; 1.79]
KIRC 1.36 [0.97; 1.90]
THCA 1.40 [0.44; 4.44]
STAD 1.41 [0.99; 2.00]
LUSC 1.42 [1.05; 1.91]
ESCA 1.51 [0.88; 2.56]
READ 5.38 [1.20; 24.17]
UVM 10.18 [2.30; 45.04]
Total 1.04 [0.88; 1.22]
Heterogeneity: χ^2_{19} = 46.47 (P < 0.001), I^2 = 59%
Test for overall effect: z = 0.43 (P = 0.67)
0.1 0.5 1 2 10
HR (95% CI)
C
PAAD
LUAD
p = 0.065
p = 0.019
group=high
group=low
D
Source HR (95% CI)
THCA 0.08 [0.01; 0.71]
KICH 0.35 [0.08; 1.51]
ACC 0.36 [0.11; 1.23]
MESO 0.39 [0.23; 0.68]
LIHC 0.43 [0.27; 0.70]
SKCM 0.50 [0.21; 1.20]
BLCA 0.51 [0.37; 0.71]
KIRP 0.53 [0.25; 1.10]
ESCA 0.57 [0.34; 0.95]
PAAD 0.65 [0.42; 1.01]
LUAD 0.66 [0.49; 0.90]
UVM 0.72 [0.29; 1.77]
BRCA 0.79 [0.54; 1.15]
STAD 0.81 [0.59; 1.13]
HNSC 0.82 [0.58; 1.15]
COAD 0.82 [0.48; 1.41]
READ 1.23 [0.37; 4.09]
LUSC 1.28 [0.96; 1.71]
KIRC 1.76 [1.29; 2.40]
CHOL 2.57 [0.83; 7.91]
Total 0.72 [0.58; 0.89]
Heterogeneity: χ^2_{19} = 72.71 (P < 0.001), I^2 = 74%
Test for overall effect: z = −3.01 (P = 0.003)
0.1 0.5 1 2 10
HR (95% CI)
E
KIRP
LIHC
p = 0.047
p < 0.0001
LUAD
PAAD
p = 0.00032
p = 0.063
group=high
group=low
F
Tip cell
R = 0.42, p = 0.2
AAT-ORR
Proportion
OV
STAD
BRCA
LUAD
Glioma
RCC
CRC
LIHC
SKCM
PRAD
PAAD
G
Veins
R = 0.017, p = 0.95
ICB-ORR
Proportion
cSCC
SKCM
CRC
NPC
RCC
LUAD
ESCA.STAD
OV
LIHC
HNSC
BRCA
Glioma
PRAD
