# Supplementary material for: Pan-cancer integrative analyses dissect the remodeling of endothelial cells in human cancers
Source: Natl Sci Rev. 2024 Jul 11;11(9):nwae231. doi: 10.1093/nsr/nwae231 (PMC11429526; doi:10.1093/nsr/nwae231)
Supplement: nwae231_Supplemental_Files [file nwae231_supplemental_files.zip › Supplementary methods.docx]

**SUPPLEMENTARY METHODS**

***Single-cell RNA-seq data collection and preprocessing***

Published scRNA-seq studies specifically focused on the TME were searched, and we only retained datasets generated by the 10X Genomics Chromium platform and without prior cell type sorting that might shift the intrinsic cellular frequencies. Available raw count matrices were downloaded and the detailed metadata were retrieved from the original studies. We performed basic preprocess and clustering analysis in each dataset separately to obtain high-quality endothelial cells. Firstly, we performed basic quality control for each dataset based on the number of detected genes per cell and the proportion of mitochondrial gene counts per cell. Specifically, cells with the number of detected genes fewer than 500 and the proportion of mitochondrial gene counts larger than 20% were filtered out. We normalized raw count matrices using the *scanpy.pp.normalize_total* function with parameter ‘*target_sum=1e4*’, and the normalized data were then logarithmically transformed for downstream analyses. Next, highly variable genes (HVGs) were selected using the *scanpy.pp.highly_variable_genes* function. To reduce data noise and reveal the main axes of variation, principal component analysis (PCA) was performed on the matrix of HVGs using the *scanpy.tl.pca* function. Following the common workflow of Scanpy [1], the adjacency matrix of the neighborhood graph was calculated using the *scanpy.pp.neighbors* function with default parameters, and Uniform Manifold Approximation and Projection (UMAP) was employed for visualization using the *scanpy.tl.umap* function. Finally, the unsupervised clustering was performed for each dataset using the *scanpy.tl.leiden* function. Endothelial cells were identified and filtered based on the specific expression of their marker genes including *PECAM1*, *PLAVP*, *VWF* and *CDH5*, and the low-to-no expression of characteristic genes expressed in other major cell types including T cells (*CD3E*, *CD3D*, *CD3G*), B cells (*CD19*, *MS4A1*), plasma cells (*MZB1*, *JCHAIN*), myeloid cells (*LYZ*, *CSF1R*, *CD68*, *CPA3*, *CSF3R*), stromal cells (*DCN*, *BGN*, *RGS5*, *ACTA2*, *MYH11*) and Epithelial cells (*EPCAM*, *KRT8*, *KRT18*).

***Data integration, batch effect correction and unsupervised clustering***

Based on the aforementioned preprocessing results, we combined the raw count matrices of endothelial cells from each dataset. We then normalized the integrated raw count matrix using the *scanpy.pp.normalize_total* function with parameter ‘*target_sum=1e4*’, and logarithmically transformed the matrix for downstream analyses. Next, the top 1000 HVGs within each dataset were re-selected using the *scanpy.pp.highly_variable_genes* function with the parameter setting ‘*batch_key=DatasetID*’, ensuring the selection of genes that are consistently present across all datasets. PCA was further performed using the *scanpy.tl.pca* function and the top 40 components were retained for downstream analyses. The BBKNN algorithm was employed to correct batch effects by identifying the closest neighbors for each cell within individual batches [2], rather than considering the entire cell pool collectively. The parameters of the *scanpy.external.pp.bbknn* function were set to ‘‘*batch_key=’DatasetID’, n_pcs=40*’’. Visualization was facilitated using the *scanpy.tl.umap* function, which employed UMAP for dimensionality reduction.

We conducted two rounds of unsupervised clustering using the *scanpy.tl.leiden* function to uncover the inherent structure within the endothelial cell population. After the first round of unsupervised clustering, each cell cluster was annotated as one of the major compartments of endothelial cells, including arteries, veins, capillaries, tip cells, or lymphatics, based on their respective signature genes. Additionally, a cluster of endothelial cells with high expression of hypoxia-related genes such as *MT1X*, *MT1E*, and *MT2A* was uniquely identified. The second round of unsupervised clustering was then performed separately within arteries, veins, capillaries, and tip cells to obtain the high-resolution map of endothelial cell populations. For each subset, the specific marker genes were identified using the *scanpy.tl.rank_genes_groups* function.

***Definition of*** ***signature gene sets***

To evaluate the functional divergence among different endothelial cell subsets, we defined three signature gene sets including angiogenesis (GO:0001525), collagen formation (R-HSA-1474290), and leukocyte-endothelial adhesion (*ICAM1*, *ICAM2*, *VCAM1*, *SELE*, *SELP*, *PECAM1*, *MADCAM1*) [3-5]. For specific endothelial cell subsets, the corresponding signature gene sets were defined as their top 8 differentially expressed genes which were calculated and ranked by the *scanpy.tl.rank_genes_groups* function. Specifically, the signature gene set of E02-tip-CXCR4 subset was defined as *CXCR4*, *ESM1*, *ANGPT2*, *INSR*, *ITGB1*, *IGFBP3*, *UNC5B* and *SPARC*, and the signature gene set of E06-veins-SELE subset was defined as *SELE*, *ICAM1*, *CCL2*, *RND1*, *NFKBIA*, *EIF1*, *SOD2*, *CXCL2*. We also used several signature gene sets that represent other cell types, including T cells (*CD3D*, *CD3E*, *CD3G*), and CD8 T cells (*CD8A*, *CD8B*).

***Calculation of signature gene score***

For scRNA-seq data, individual cells were scored using the *decoupler.run_aucell* function [6], which uses the Area Under the Curve (AUC) to calculate whether a set of targets is enriched within the molecular readouts of each sample. For the calculation of average signature scores of major compartments or subpopulations, we randomly select an equal number of cells from each cancer type to calculate mean scores, thereby avoiding the impact of varying endothelial cell counts across different cancer types on the calculation of averages. In particular, we randomly sampled 2000 cells per cancer type, at which point the variance of scores across multiple replicates was less than 0.001. For bulk RNA-seq and microarray data, each sample was scored using the “ssgsea” method of R package GSVA with parameters setting “*kcdf=’Gaussian’, ssgsea.norm=T*” [7].

***Prediction of cytokine signaling activity***

We utilized the CytoSig prediction model to examine the activity of cytokine signaling [8]. The activity score of each cytokine signaling to each cell was first calculated using the *CytoSig.ridge_significance_test* function with parameters setting “*alpha = 1E4, nrand = 1000, alternative='two-sided’*”, and the activity score of each cytokine signaling to each endothelial cell subset was then defined as the average score of corresponding cells. As we mentioned above, we randomly select an equal number of cells from each cancer type to calculate mean scores, thereby avoiding the impact of varying endothelial cell counts across different cancer types on the calculation of averages. In addition, the top 25 cytokines, sorted by absolute activity score values, were used for visualization.

***Vizgen MERFISH data preprocessing and analysis***

We obtained the public MERFISH data, including cell-by-gene matrix and cell-metadata file through the Data Release Program of vizgen [9]. The AnnData object was constructed by the python package Squidpy [10], and cells with counts fewer than 50 and genes that were expressed in fewer than 10 cells were filtered. We normalized and logarithmically transformed the raw data using the *scanpy.pp.normalize_total* and the *scanpy.pp.log1p* function, respectively. The gene expression was scaled to unit variance using the *scanpy.pp.scale* function following the pipeline of Squdipy. Next, the *scanpy.tl.pca* function was employed to reduce the dimensionality of data, and the neighborhood graph of cells was further calculated on PCA space using the *sc.pp.neighbors* with parameters setting “*n_neighbors=10, n_pcs=20*”. We visualized all cells in a two-dimensional UMAP embedding using *the scanpy.tl.umap* function and performed unsupervised clustering using the *scanpy.tl.leiden* function. The major compartments of cells were annotated based on their available marker genes, with endothelial cells featuring specific expression of *PECAM1*. Endothelial cells were extracted and performed the second-round unsupervised clustering. Despite the limited number of genes in the MERFISH data, we were still able to consistently identify *CXCR4*^+^ tip cells and *ICAM1*^+^ veins, representing E02-tip-CXCR4 and E06-veins-SELE respectively. Notably, the latter also highly expressed *CCL2*, another marker gene of E06-veins-SELE, while *SELE* was not included in the MEFISH gene panel. For the presentation of global views and ROI examples, we showcased the distribution patterns of T cells, epithelial/malignant cells, *CXCR4*^+^ tip cells, and *ICAM1*^+^ veins using the *squidpy.pl.spatial_scatter* function.

We assessed the spatial distribution characteristics of *CXCR4*^+^ tip cells and *ICAM1*^+^ veins using two distinct approaches. Initially, we performed 400 random local region samplings for each MERFISH data slice, with each sampling area covering a 500$\times$500 unit area. Within each sampled local region, we tallied the numbers of *CXCR4*^+^ tip cells and *ICAM1*^+^ veins, categorizing these regions based on their respective counts. Subsequently, we categorized these regions into *CXCR4*^+^ tip cell high/low or *ICAM1*^+^ veins high/low groups, based on the quantity of *CXCR4*^+^ tip cells and *ICAM1*^+^ veins respectively. We then counted the numbers of T cells and epithelial/malignant cells within the regions categorized and performed statistical analyses based on these groupings. For the second approach, we computed the spatial connectivity graph for each whole MERFISH slice using the *squidpy.gr.spatial_neighbors* function, calculating the cellular compositions of spatial neighbors of *CXCR4*^+^ tip cells and *ICAM1*^+^ veins. We then performed chi-square tests for the counts of T cells and epithelial/malignant cells that constituted spatial neighbors of *CXCR4*^+^ tip cells and *ICAM1*^+^ veins, respectively, and calculated the corresponding Pearson residuals.

***Evaluation of the endothelial cell similarity among cancer types***

To assess the similarity of each major compartment of endothelial cells among various cancers, we first extracted the average gene expression vectors of each major compartment in each cancer type, and only tumor tissue-derived endothelial cells were enrolled in this analysis. The Pearson correlation coefficients of all major compartments of endothelial cells among cancers were then obtained respectively based on their transcriptome. We used the *radarchart* plot function of R package fmsb to visualize the results.

***Hierarchical clustering of cancer types***

For the comparison of the endothelial cell compositions across various cancer types, we conducted unsupervised hierarchical clustering for the cancer types based on their respective subset proportions within all endothelial cells. The frequencies of all subsets served as input for the R function *tree* to hierarchically cluster the different cancer types. Specifically, only endothelial cells derived from tumors and the cancer type with tumor-derived samples >3 were utilized for this analysis.

***Tissue enrichment analysis of endothelial cell subsets***

We assessed the tissue preference of each endothelial cell subset across different tissues by calculating their Pearson residuals. Specifically, for the adjacent non-tumor and tumor tissue paired samples, the number of each endothelial cell subset in these two tissues was tallied. We then obtained the Pearson residual of each endothelial cell subset in tumor tissue using the chi-square test. A subset was considered enriched in tumor tissue if the corresponding Pearson residual value was greater than 0 and depleted if the corresponding Pearson residual value was less than 0. The absolute magnitude of the Pearson residual value can reflect the extent of enrichment or depletion.

***Differential gene expression analysis***

To identify differentially expressed genes between venous endothelial cells in tumors and those in adjacent non-tumor tissues, we first calculated the significance of each gene by two-sided unpaired Wilcoxon test with multiple hypothesis correction using the Benjamini-Hochberg procedure. Genes with adjusted p*-*values less than 0.05 were identified as differentially expressed genes. Additionally, log_2_(FC) was calculated by subtracting log2-transformed mean counts in tumor tissue to those in adjacent non-tumor tissue.

In addition, to identify consistent gene expression changes across major compartments of endothelial cells, we first performed differential gene expression analyses between non-tumor and tumor tissues for veins, arteries, capillaries and tip cells, separately. Subsequently, we obtained top 400 upregulated genes in tumors compared to non-tumor tissues for each major compartment. The upset plot was utilized to visualize intersections. To further pinpoint cancer type-conserved TEC signatures, we acquired top 400 upregulated genes in TECs compared to endothelial cells from non-tumor tissues, for each cancer type separately. We then screened these genes to ensure their significant upregulation in at least 10 cancer types, and mean rank scores surpassing 10, aiming to identify robust TEC signatures. Finally, we identified seven consistently upregulated genes shared across cancer types and cellular compartments. Furthermore, for endothelial cells of each cancer type, we computed the top 400 upregulated genes relative to endothelial cells of other cancer types. The pathway enrichment analysis was then conducted based on these upregulated genes to obtain enriched Gene Ontology pathways for endothelial cells of each cancer type.

***TCGA data analysis***

The expression matrix of TCGA and the corresponding clinical metadata of samples were downloaded from the TCGA Pan-Cancer cohort on the UCSC Xena website (<https://xenabrowser.net/>). We only used samples from primary tumors and excluded non-solid cancer types since we focused on endothelial cells. First, the signature scores of the T cell and CD8 T cell, as well as the E02-tip-CXCR4 subset and E06-veins-SELE subset were calculated using the “ssgsea” method from the R package GSVA as aforementioned. We then performed correlation analyses between the T/CD8 T cell score and E06-veins-SELE subset score of each sample within each analyzed cancer type.

For the tumor stage-related analyses, the samples without information about the tumor stage were removed, and we aggregated original tumor stage annotations into stages I, II, III, and IV. For example, stages IA and IB were grouped as stage I, while stages IIA, IIB, and IIC were grouped as stage II. We then combined stages I and II as the early stage, and stages III and IV as the late stage. Next, the mean values of the E02-tip-CXCR4 subset score and the E06-veins-SELE subset score were calculated respectively for samples belonging to the same stage within each analyzed cancer type. The corresponding standard error of the mean (SEM) value was calculated by the *summarySE* function of the R package Rmisc.

For survival analyses, we used the overall survival (OS) as the endpoint to evaluate the clinical outcomes of patients. Notably, given the distinct preference of the two subsets, E02-tip-CXCR4 and E06-veins-SELE, for different tumor stages, we still opted to exclude patients without tumor stage information. The effect of the tumor stage was corrected in the subsequent Cox proportional hazards model to ensure that the observed signals were unaffected by the variations in the tumor stage. Specifically, the signature score of E02-tip-CXCR4 was defined as above, and the relative enrichment score of E06-veins-SELE was defined as the signature score of E06-veins-SELE divided by the signature score of E02-tip-CXCR4 for each patient. To explore the effects of these signatures on overall survival, the TCGA patients within each cancer type were classified into high and low groups based on the optimal cutpoint returned by the *surv_cutpoint* function with the parameter setting “*minprop=0.3*”. Survival analyses were then performed by the Cox proportional hazards model implemented in the R package survival, after correcting other clinical factors including tumor stage, gender, and age. Next, we fitted survival curves using the Kaplan-Meier formula in the *survfit* function, and visualized curves using the *ggsurvplot* function of the R package survminer. For analyzed cancer types, the meta-analysis of Hazard ratio was calculated using the *metagen* function of the R package meta, and visualized using the *forest* function. In addition, we performed deep learning–based cell composition analysis to further validate the association between the enrichment score of E06-veins-SELE and prognosis based on Scaden [11]. We trained the neural network model on simulated bulk samples which were generated by randomly combining different numbers of cell types. We then applied the optimized model on TCGA data and performed the survival analyses as we mentioned above based on the deconvoluted results.

***Analysis of ICB and AAT datasets***

Public bulk RNA-seq datasets of ICB therapy [12-15] were collected and only the baseline samples were used for analyses. First, all count matrices were normalized (TPM) and log2-transformed. We then renamed patients as responder (including CR/PR patients) or non-responder (including SD/PD patients) based on their original clinical responses after ICB treatment. As for AAT data, we collected the processed matrices and metadata profiles from CTR-db, a web-based database specifically designed to comprehensively collect and uniformly reprocess patient-derived clinical transcriptomes related to cancer drug response [16]. Due to a limited number of patients receiving other anti-angiogenesis drugs, we exclusively utilized the data pertaining to the Bevacizumab treatment. Finally, for patients of each dataset, we calculated the enrichment score of E06-veins-SELE relative to E02-tip-CXCR4 using the “ssgsea” method of R package GSVA as aforementioned, and further compared scores between responders and non-responders. For the public scRNA-seq datasets of ICB therapy [17, 18], we performed a similar preprocess pipeline as we described earlier. Following unsupervised clustering, the cell populations represented E02-tip-CXCR4 and E06-veins-SELE cells were identified, and we then obtained and compared their frequencies between responders and non-responders.

***Correlation analysis of subset proportion and ORR***

The relationship between the proportions of endothelial cell subsets and the response to different therapy strategies in varied cancer types was evaluated. For ICB therapy, we collected previously reported ORR data of anti-PD-1/PD-L1 treatment from the study by Yarchoan et al [19]. For AATs, we manually gathered the ORR data of anti-VEGF treatment reported in previous large cohort clinical trials or meta-analyses, including in CRC [20], LUAD [21], STAD [22], PRAD [23], PAAD [24], SKCM [25], BRCA [26], LIHC [27], RCC [28], OV [29], and glioma [30]. We then performed correlation analyses between the proportions of endothelial cell subsets and ORR value across cancer types.

***Statistical analysis***

Statistical analyses used in this study included the Kruskal-Wallis test, Wilcoxon test, hypergeometric test, chi-square test, t-test, and log-rank test as described in the Figure legends. The Kaplan-Meier method was applied in survival analyses.

# Reference

1. Wolf FA, Angerer P, Theis FJ. SCANPY: large-scale single-cell gene expression data analysis. *Genome Biology*. 2018; **19**(1): 15. doi: 10.1186/s13059-017-1382-0

2. Polański K, Young MD, Miao Z *et al.* BBKNN: fast batch alignment of single cell transcriptomes. *Bioinformatics*. 2019; **36**(3): 964-965. doi: 10.1093/bioinformatics/btz625 %J Bioinformatics

3. Panés J, Perry M, Granger DN. Leukocyte-endothelial cell adhesion: avenues for therapeutic intervention. *British journal of pharmacology*. 1999; **126**(3): 537-550. doi: 10.1038/sj.bjp.0702328

4. Langer HF, Chavakis T. Leukocyte-endothelial interactions in inflammation. *Journal of cellular and molecular medicine*. 2009; **13**(7): 1211-1220. doi: 10.1111/j.1582-4934.2009.00811.x

5. Xue R, Zhang Q, Cao Q *et al.* Liver tumour immune microenvironment subtypes and neutrophil heterogeneity. *Nature*. 2022; **612**(7938): 141-147. doi: 10.1038/s41586-022-05400-x

6. Badia-i-Mompel P, Vélez Santiago J, Braunger J *et al.* decoupleR: ensemble of computational methods to infer biological activities from omics data. *Bioinformatics Advances*. 2022; **2**(1). doi: 10.1093/bioadv/vbac016

7. Hänzelmann S, Castelo R, Guinney J. GSVA: gene set variation analysis for microarray and RNA-Seq data. *BMC Bioinformatics*. 2013; **14**(1): 7. doi: 10.1186/1471-2105-14-7

8. Jiang P, Zhang Y, Ru B *et al.* Systematic investigation of cytokine signaling activity at the tissue and single-cell levels. *Nature Methods*. 2021; **18**(10): 1181-1191. doi: 10.1038/s41592-021-01274-5

9. Vizgen MERFISH FFPE Human Immuno-oncology Data Set. 2022.

10. Palla G, Spitzer H, Klein M *et al.* Squidpy: a scalable framework for spatial omics analysis. *Nat Methods*. 2022; **19**(2): 171-178. doi: 10.1038/s41592-021-01358-2

11. Menden K, Marouf M, Oller S *et al.* Deep learning-based cell composition analysis from tissue expression profiles. *Science advances*. 2020; **6**(30): eaba2619. doi: 10.1126/sciadv.aba2619

12. Cho J-W, Hong MH, Ha S-J *et al.* Genome-wide identification of differentially methylated promoters and enhancers associated with response to anti-PD-1 therapy in non-small cell lung cancer. *Experimental & Molecular Medicine*. 2020; **52**(9): 1550-1563. doi: 10.1038/s12276-020-00493-8

13. Hugo W, Zaretsky JM, Sun L *et al.* Genomic and Transcriptomic Features of Response to Anti-PD-1 Therapy in Metastatic Melanoma. *Cell*. 2016; **165**(1): 35-44. doi: <https://doi.org/10.1016/j.cell.2016.02.065>

14. He Y, Ramesh A, Gusev Y *et al.* Molecular predictors of response to pembrolizumab in thymic carcinoma. *Cell Reports Medicine*. 2021; **2**(9): 100392. doi: <https://doi.org/10.1016/j.xcrm.2021.100392>

15. Choueiri TK, Fishman MN, Escudier B *et al.* Immunomodulatory Activity of Nivolumab in Metastatic Renal Cell Carcinoma. *Clinical cancer research : an official journal of the American Association for Cancer Research*. 2016; **22**(22): 5461-5471. doi: 10.1158/1078-0432.Ccr-15-2839

16. Liu Z, Liu J, Liu X *et al.* CTR-DB, an omnibus for patient-derived gene expression signatures correlated with cancer drug response. *Nucleic Acids Research*. 2022; **50**(D1): D1184-D1199. doi: 10.1093/nar/gkab860

17. Yost KE, Satpathy AT, Wells DK *et al.* Clonal replacement of tumor-specific T cells following PD-1 blockade. *Nat Med*. 2019; **25**(8): 1251-1259. doi: 10.1038/s41591-019-0522-3

18. Li J, Wu C, Hu H *et al.* Remodeling of the immune and stromal cell compartment by PD-1 blockade in mismatch repair-deficient colorectal cancer. *Cancer Cell*. 2023; **41**(6): 1152-1169.e1157. doi: 10.1016/j.ccell.2023.04.011

19. Yarchoan M, Albacker LA, Hopkins AC *et al.* PD-L1 expression and tumor mutational burden are independent biomarkers in most cancers. *JCI Insight*. 2019; **4**(6). doi: 10.1172/jci.insight.126908

20. Qu CY, Zheng Y, Zhou M *et al.* Value of bevacizumab in treatment of colorectal cancer: A meta-analysis. *World journal of gastroenterology*. 2015; **21**(16): 5072-5080. doi: 10.3748/wjg.v21.i16.5072

21. Liu Y, Li HM, Wang R. Effectiveness and Safety of Adding Bevacizumab to Platinum-Based Chemotherapy as First-Line Treatment for Advanced Non-Small-Cell Lung Cancer: A Meta-Analysis. *Frontiers in medicine*. 2021; **8**: 616380. doi: 10.3389/fmed.2021.616380

22. Ohtsu A, Shah MA, Van Cutsem E *et al.* Bevacizumab in combination with chemotherapy as first-line therapy in advanced gastric cancer: a randomized, double-blind, placebo-controlled phase III study. *Journal of clinical oncology : official journal of the American Society of Clinical Oncology*. 2011; **29**(30): 3968-3976. doi: 10.1200/jco.2011.36.2236

23. Qi WX, Fu S, Zhang Q *et al.* Efficacy and toxicity of anti-VEGF agents in patients with castration-resistant prostate cancer: a meta-analysis of prospective clinical studies. *Asian Pacific journal of cancer prevention : APJCP*. 2014; **15**(19): 8177-8182. doi: 10.7314/apjcp.2014.15.19.8177

24. Kindler HL, Niedzwiecki D, Hollis D *et al.* A double-blind, placebo-controlled, randomized phase III trial of gemcitabine (G) plus bevacizumab (B) versus gemcitabine plus placebo (P) in patients (pts) with advanced pancreatic cancer (PC): A preliminary analysis of Cancer and Leukemia Group B (CALGB. *Journal of Clinical Oncology*. 2007; **25**(18_suppl): 4508-4508. doi: 10.1200/jco.2007.25.18_suppl.4508

25. Han X, Ge P, Liu S *et al.* Efficacy and safety of bevacizumab in patients with malignant melanoma: a systematic review and PRISMA-compliant meta-analysis of randomized controlled trials and non-comparative clinical studies. *Frontiers in pharmacology*. 2023; **14**: 1163805. doi: 10.3389/fphar.2023.1163805

26. Miller K, Wang M, Gralow J *et al.* Paclitaxel plus bevacizumab versus paclitaxel alone for metastatic breast cancer. *The New England journal of medicine*. 2007; **357**(26): 2666-2676. doi: 10.1056/NEJMoa072113

27. Finn RS, Qin S, Ikeda M *et al.* IMbrave150: Updated overall survival (OS) data from a global, randomized, open-label phase III study of atezolizumab (atezo) + bevacizumab (bev) versus sorafenib (sor) in patients (pts) with unresectable hepatocellular carcinoma (HCC). *Journal of Clinical Oncology*. 2021; **39**(3_suppl): 267-267. doi: 10.1200/JCO.2021.39.3_suppl.267

28. Escudier BJ, Bellmunt J, Negrier S *et al.* Final results of the phase III, randomized, double-blind AVOREN trial of first-line bevacizumab (BEV) + interferon-α2a (IFN) in metastatic renal cell carcinoma (mRCC). *Journal of Clinical Oncology*. 2009; **27**(15_suppl): 5020-5020. doi: 10.1200/jco.2009.27.15_suppl.5020

29. Perren TJ, Swart AM, Pfisterer J *et al.* A phase 3 trial of bevacizumab in ovarian cancer. *The New England journal of medicine*. 2011; **365**(26): 2484-2496. doi: 10.1056/NEJMoa1103799

30. Wick W, Gorlia T, Bendszus M *et al.* Lomustine and Bevacizumab in Progressive Glioblastoma. *The New England journal of medicine*. 2017; **377**(20): 1954-1963. doi: 10.1056/NEJMoa1707358

**Supplementary tables**

Table S1. Dataset information.

Table S2. Differentially expressed genes for major compartments and subsets of endothelial cells.

Table S3. Detailed Gene Ontology pathways enriched in the TECs of each cancer type.

Table S4. Upregulated genes of blood vascular endothelial cells in tumor tissue.

Table S5. Upregulated genes of TECs in different cancer types.

Table S6. Differentially expressed genes between tumor and adjacent non-tumor tissues for veins.
