# Supplementary figures and images for: Pan-cancer integrative analyses dissect the remodeling of endothelial cells in human cancers
Source: Natl Sci Rev. 2024 Jul 11;11(9):nwae231. doi: 10.1093/nsr/nwae231 (PMC11429526; doi:10.1093/nsr/nwae231)

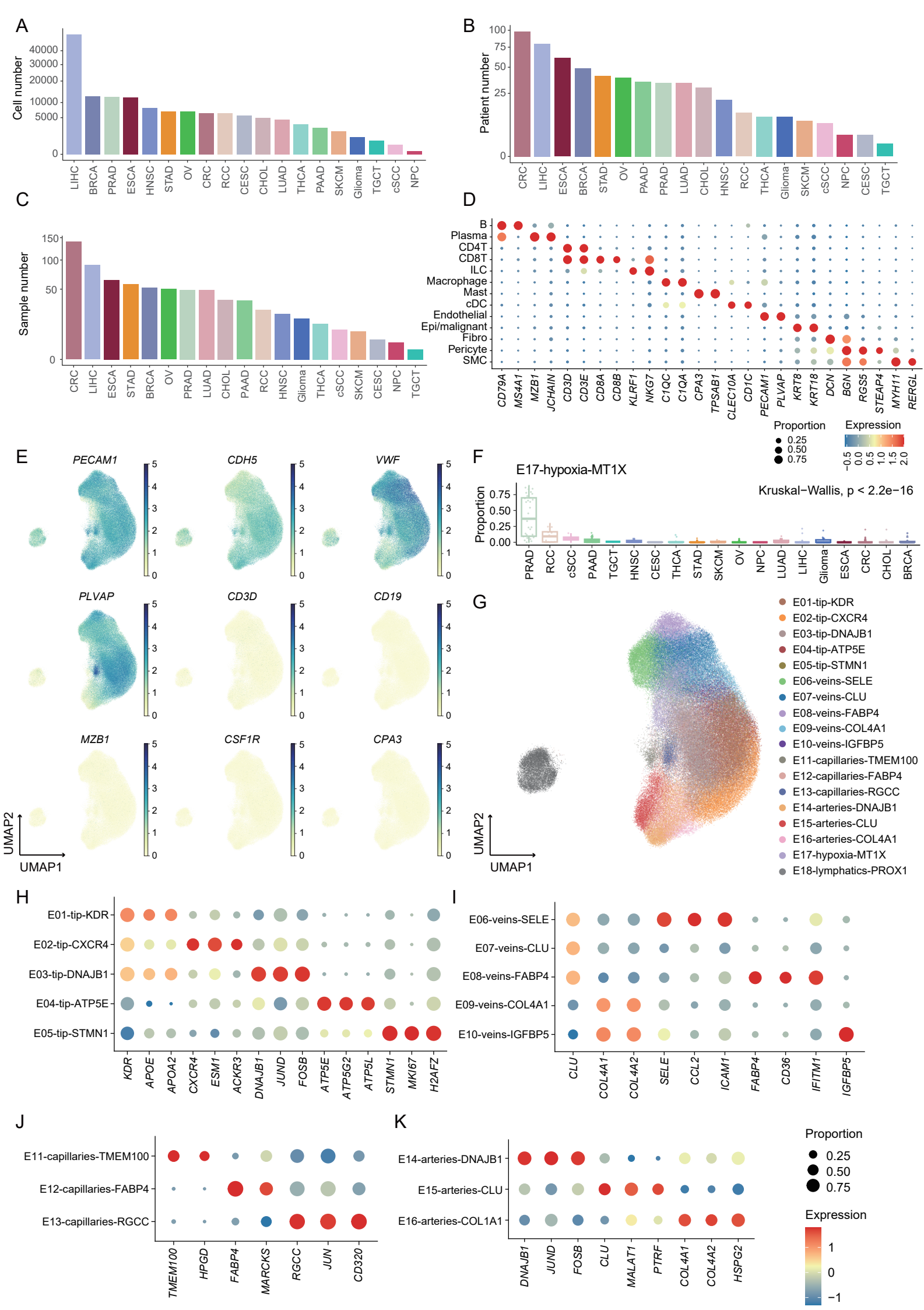

Supplement: nwae231_Supplemental_Files [file nwae231_supplemental_files.zip › Supplementary figures/sfig1.pdf]

A

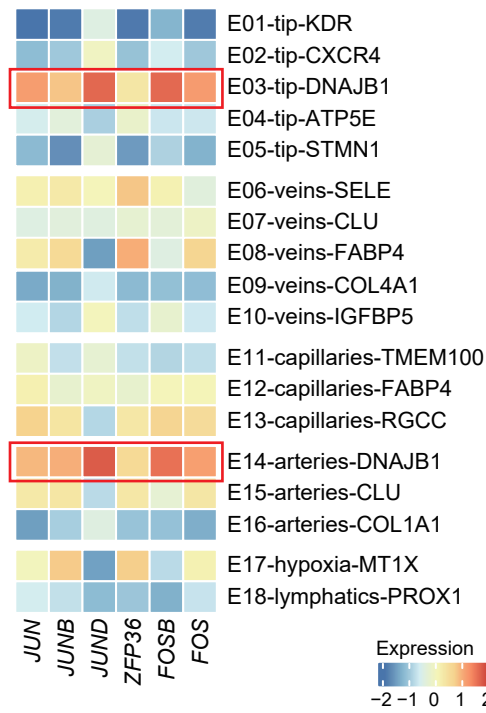

B

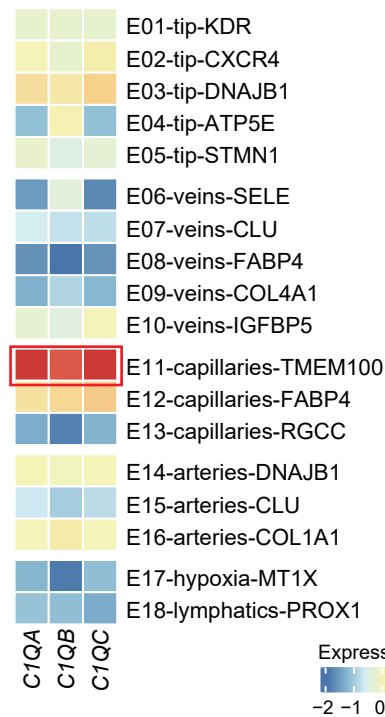

C

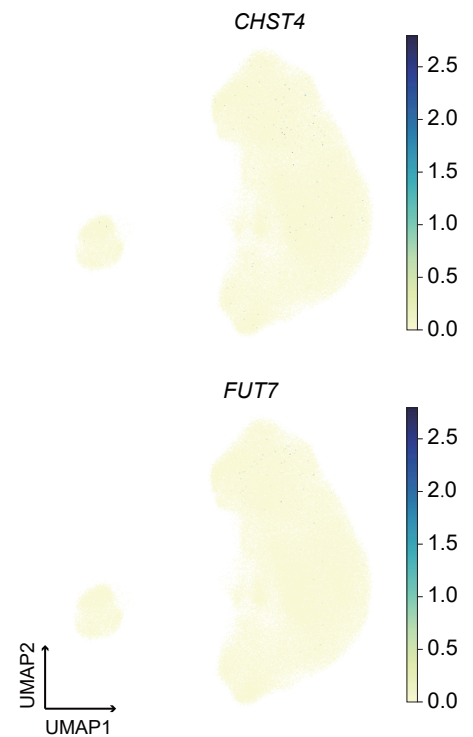

D

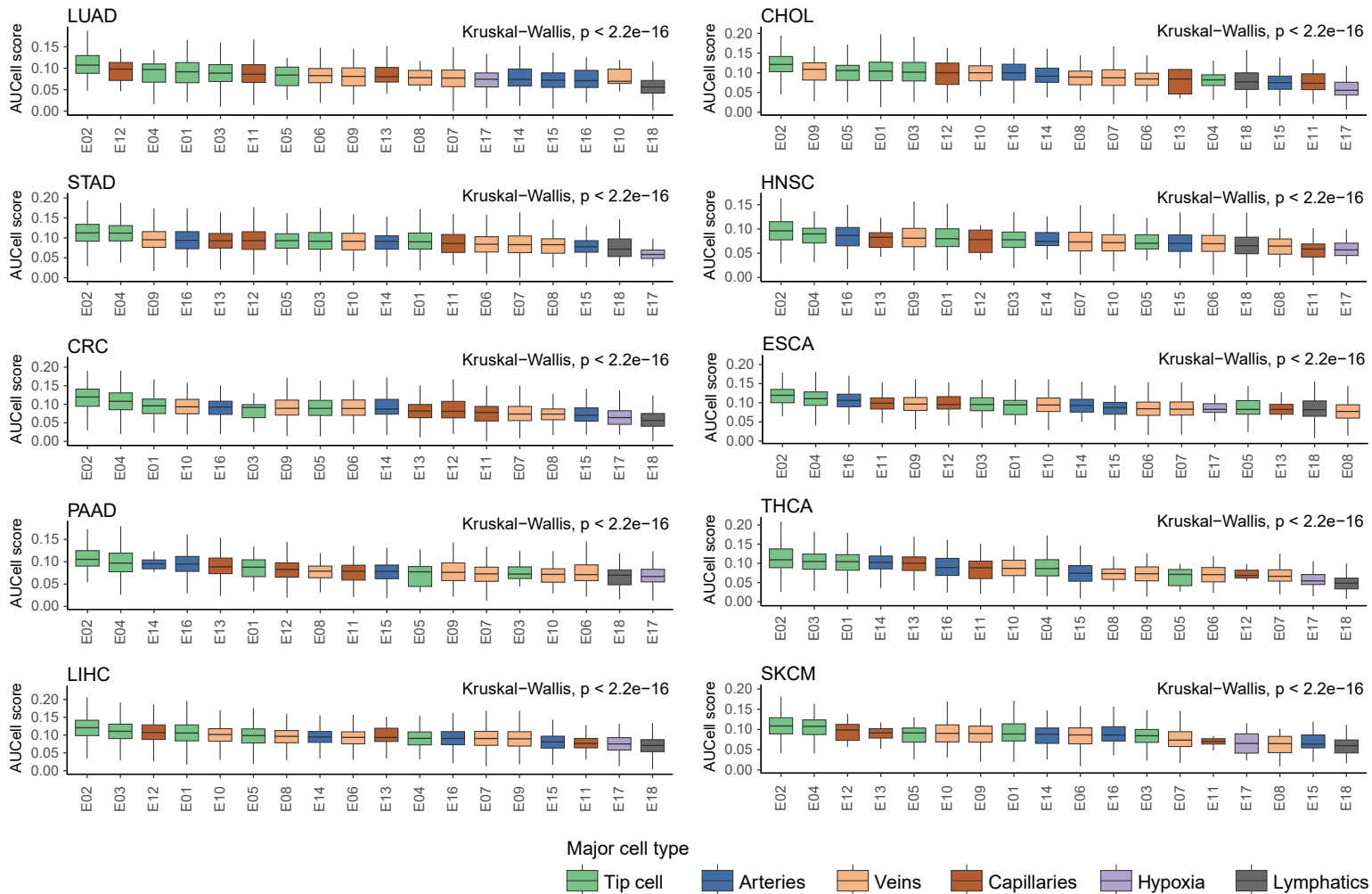

Supplement: nwae231_Supplemental_Files [file nwae231_supplemental_files.zip › Supplementary figures/sfig2.pdf]

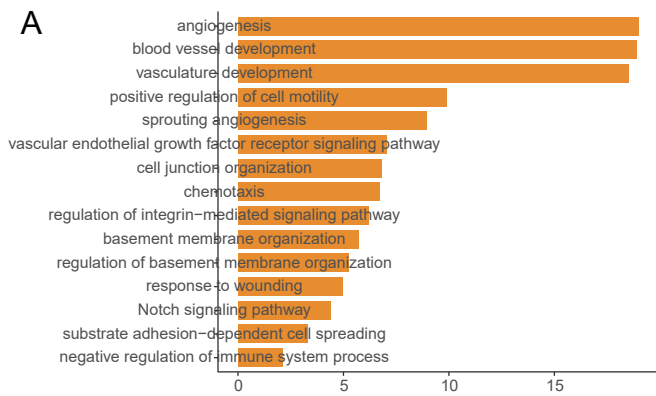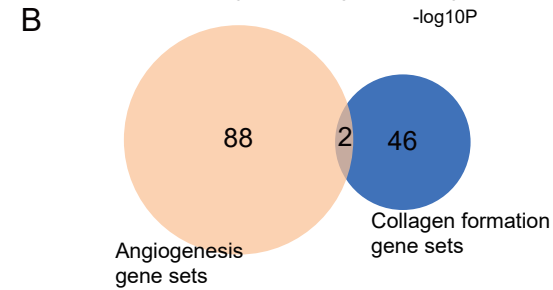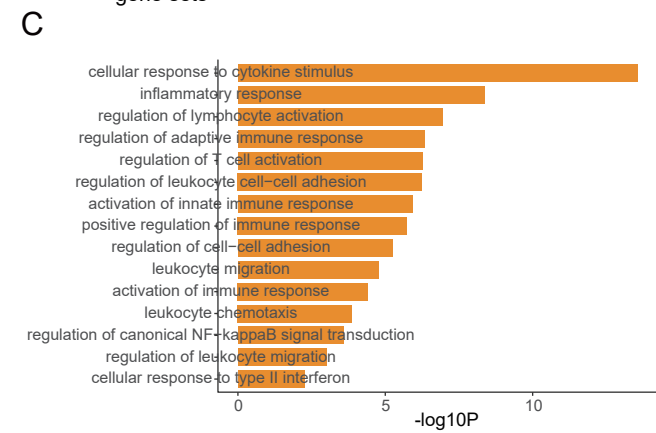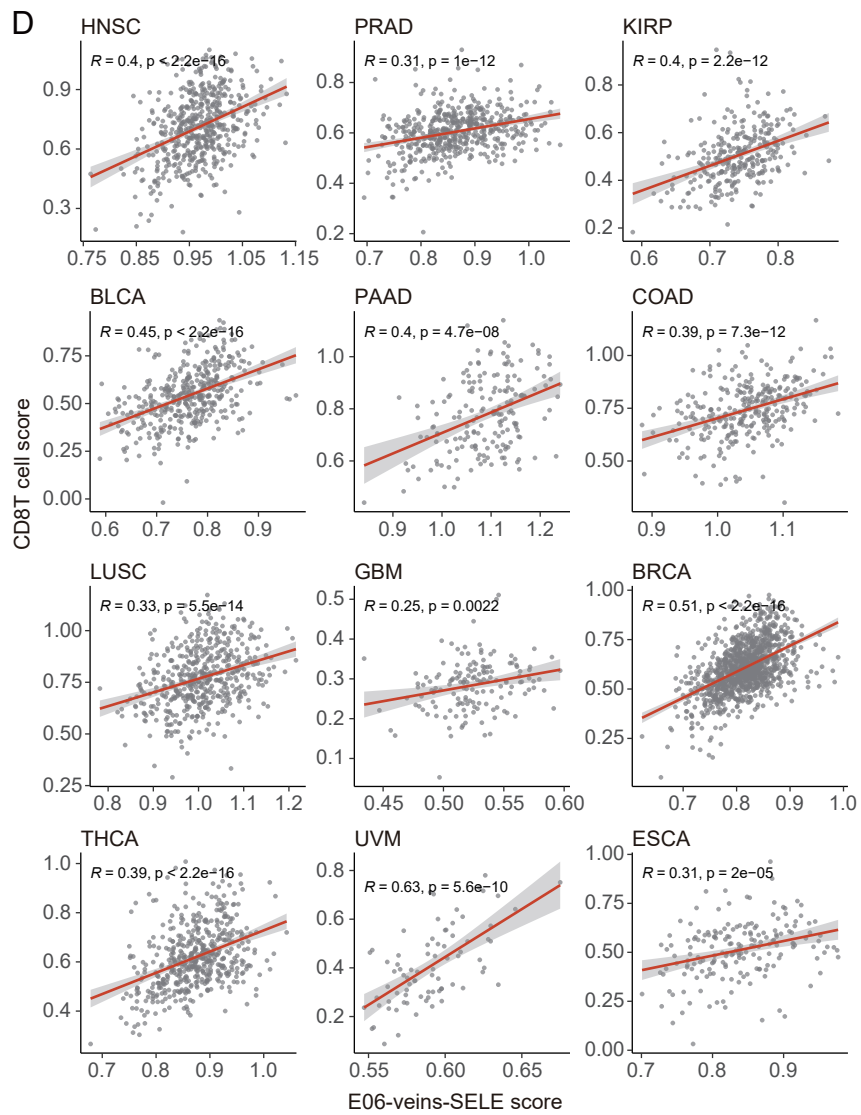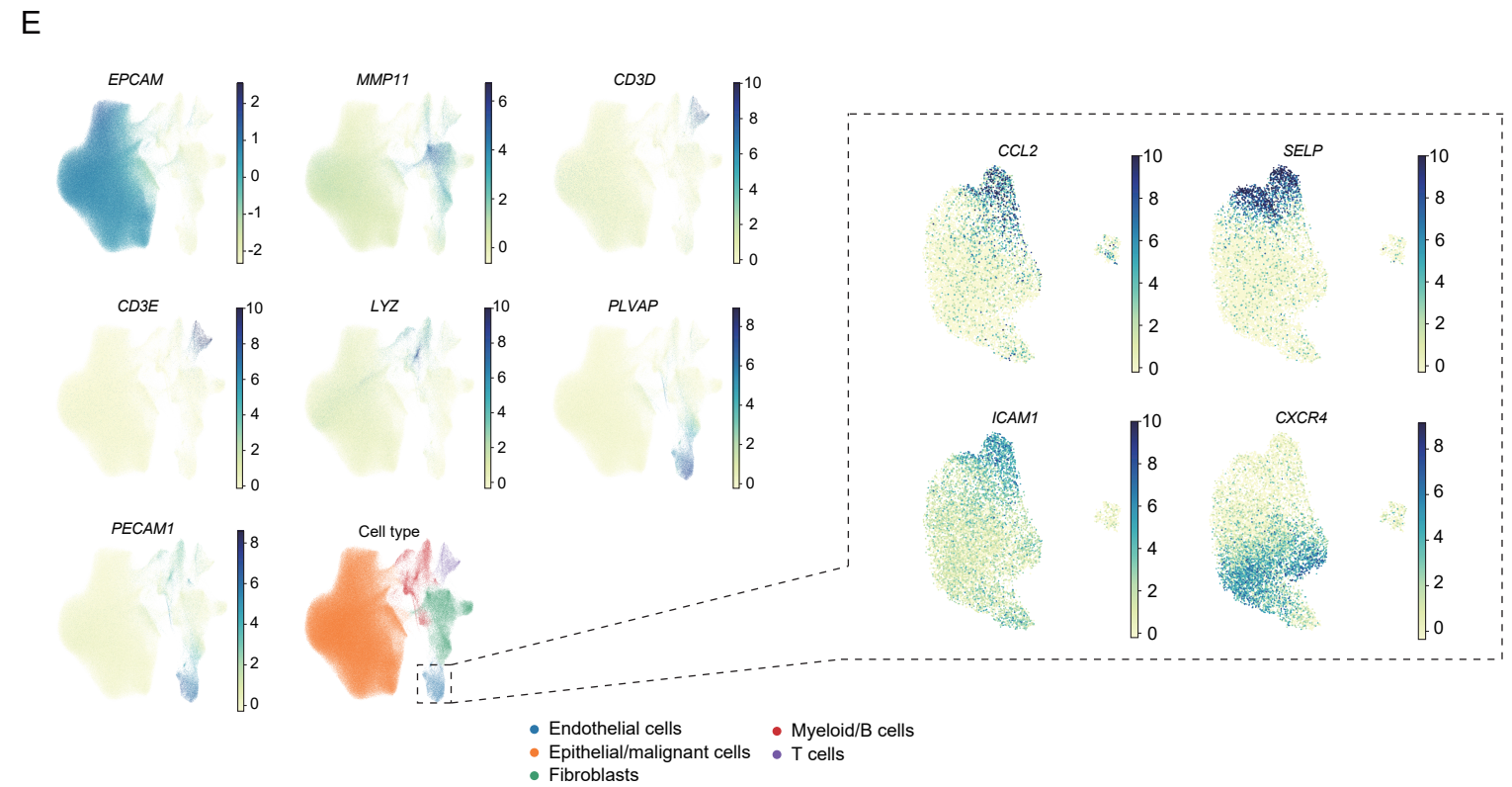

Supplement: nwae231_Supplemental_Files [file nwae231_supplemental_files.zip › Supplementary figures/sfig3.pdf]

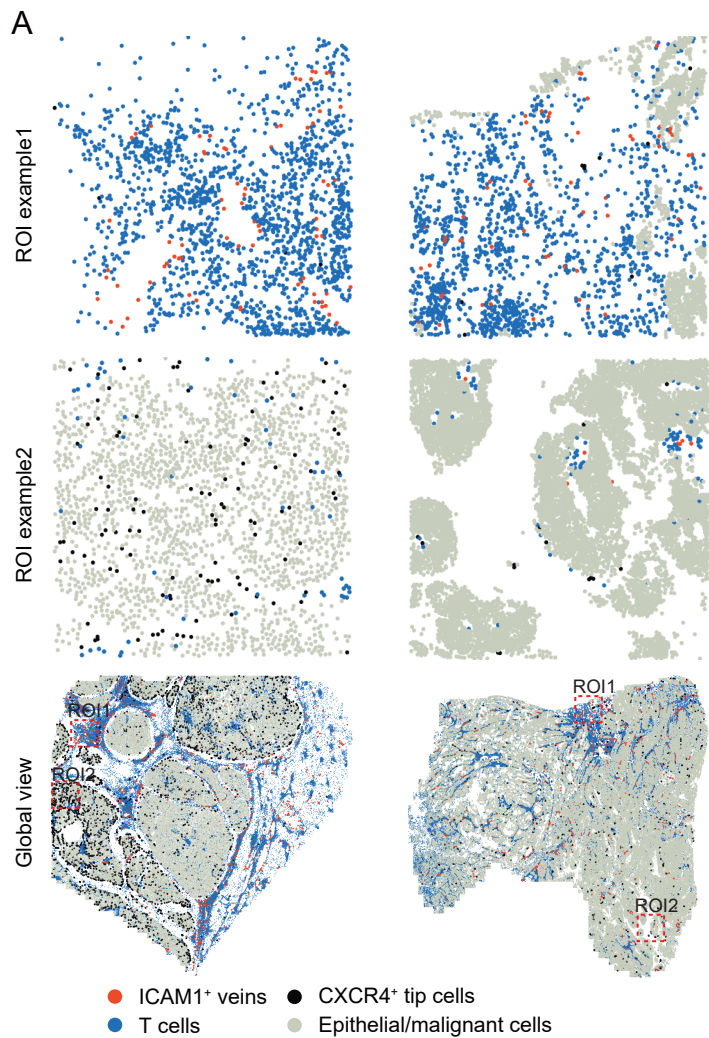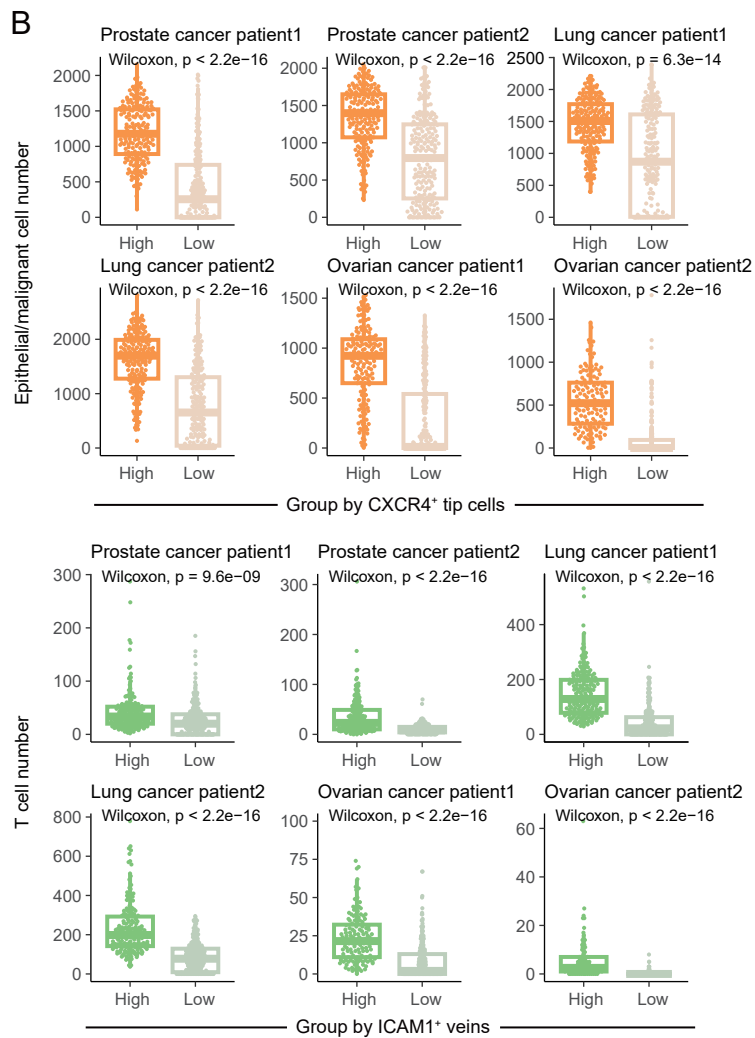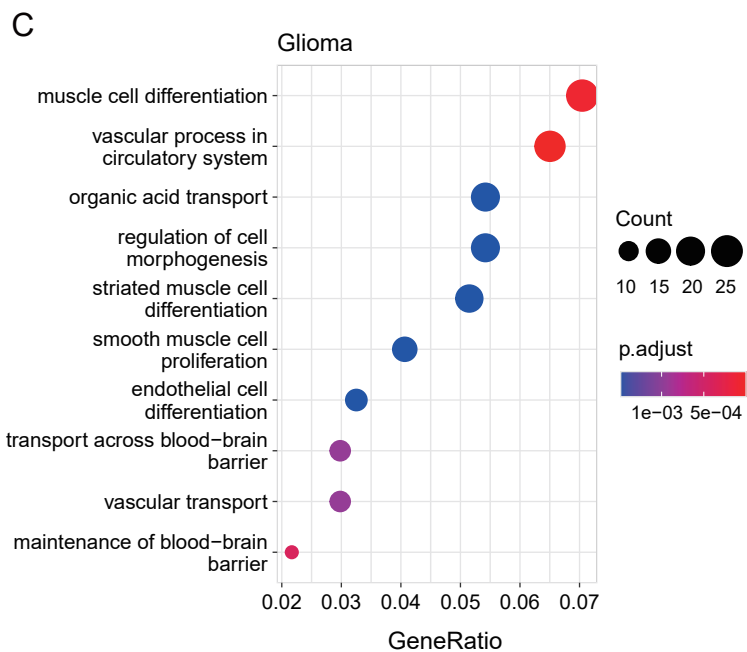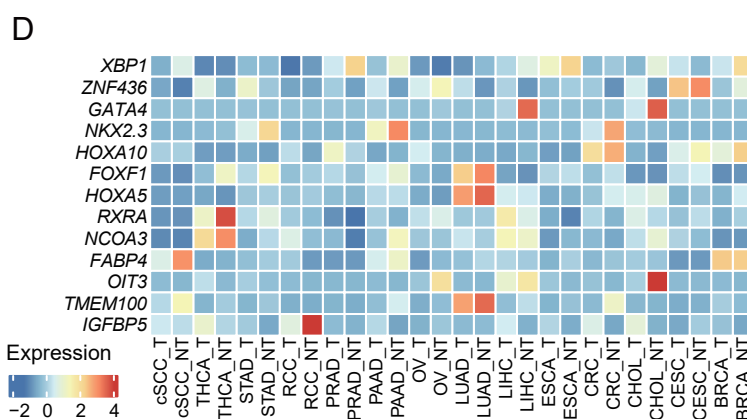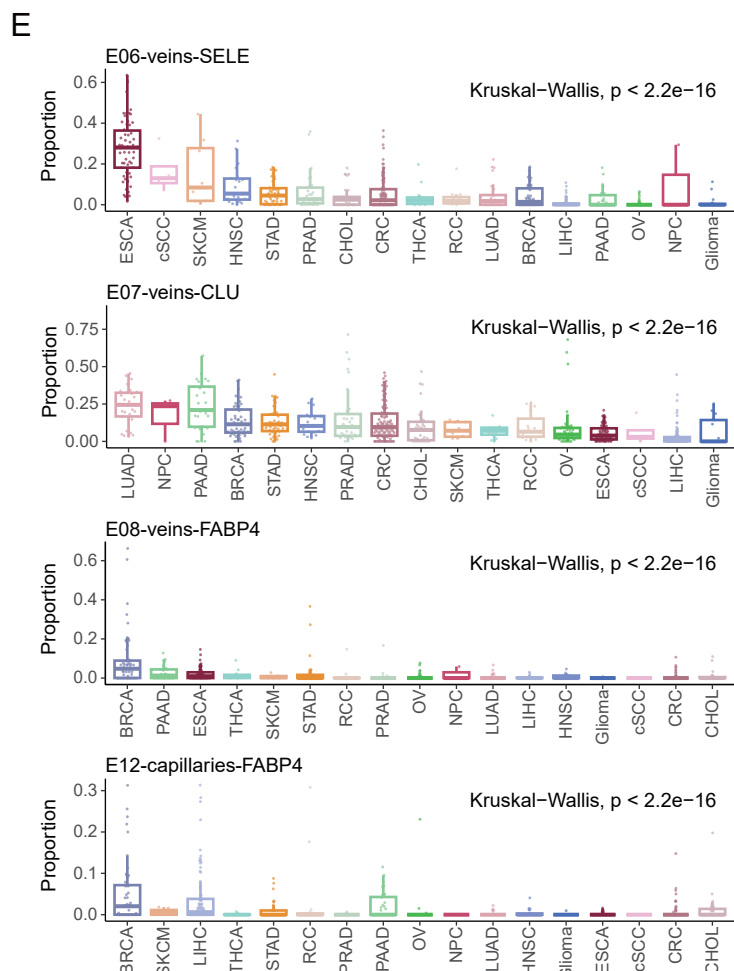

Supplement: nwae231_Supplemental_Files [file nwae231_supplemental_files.zip › Supplementary figures/sfig4.pdf]

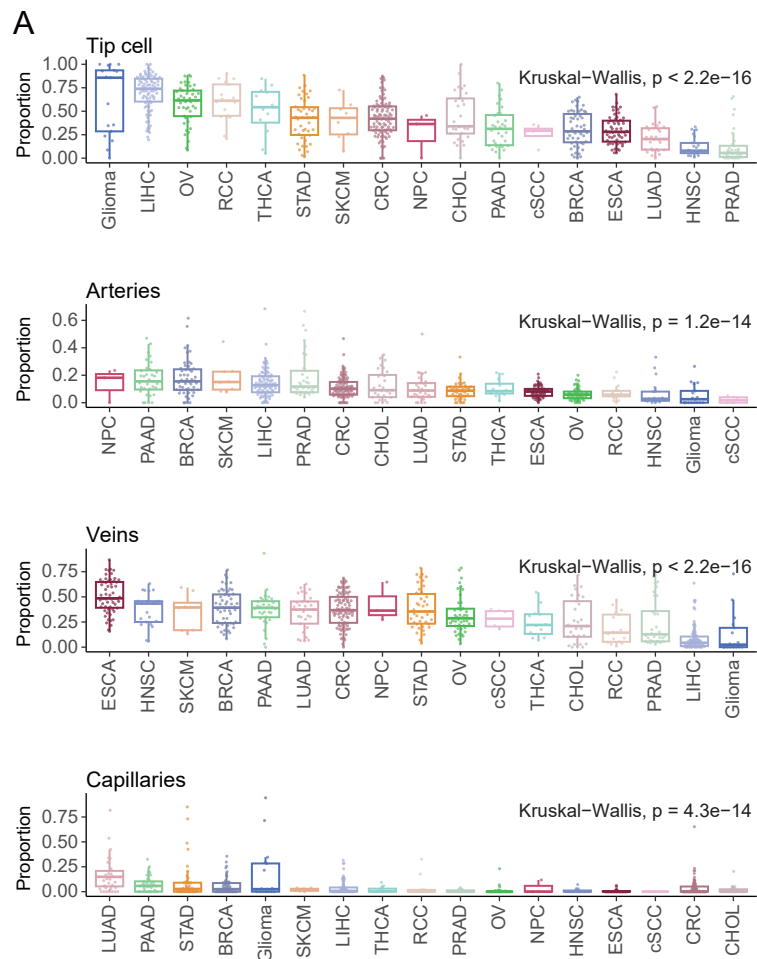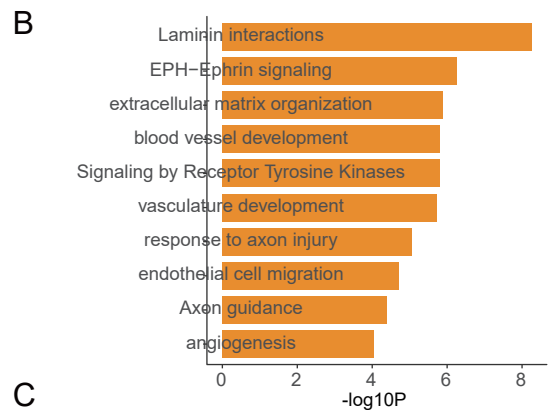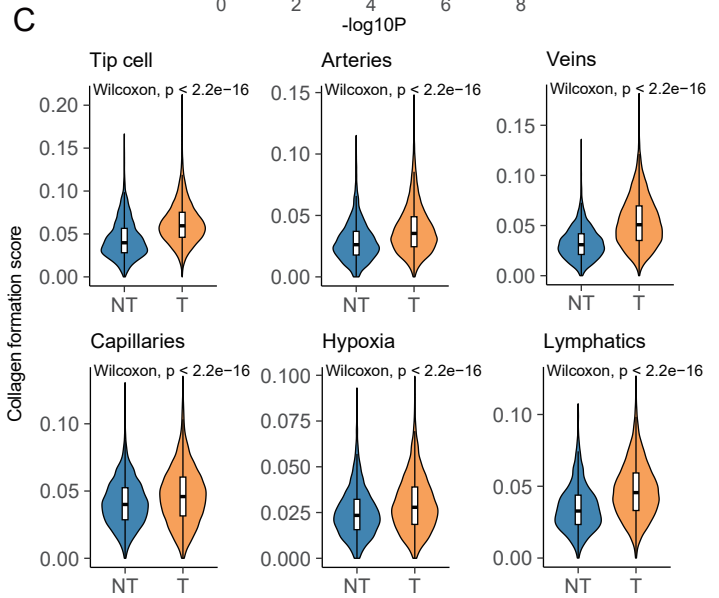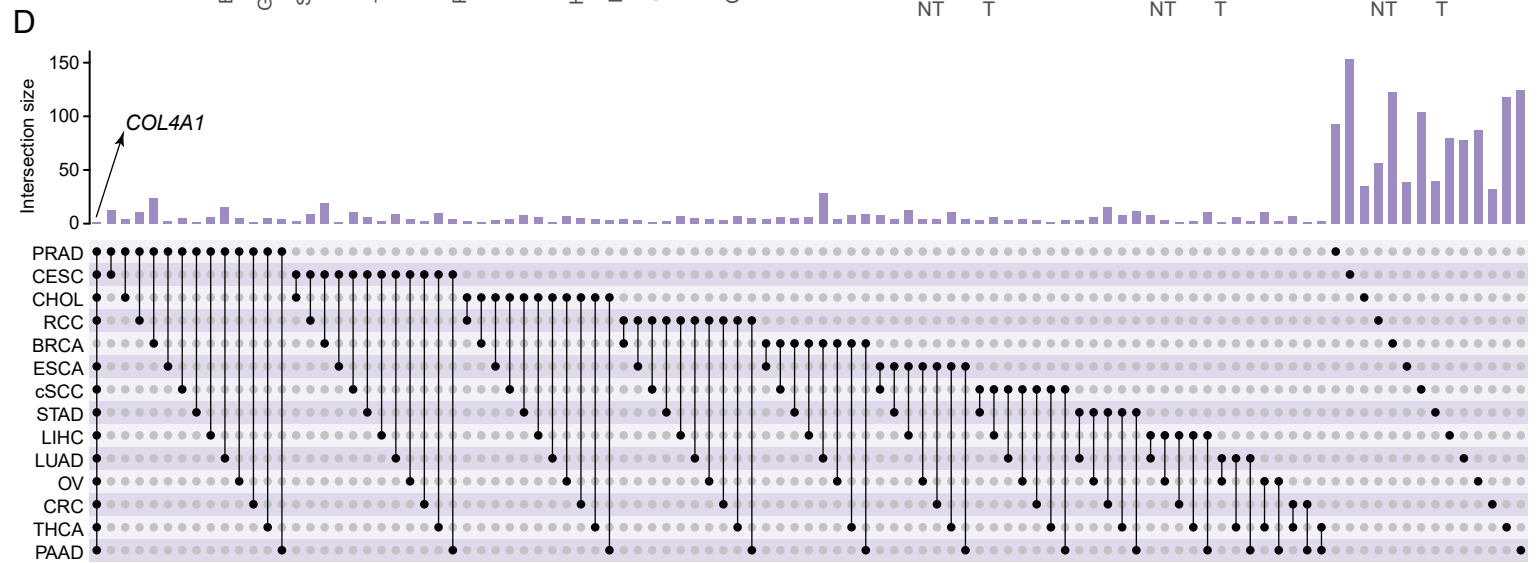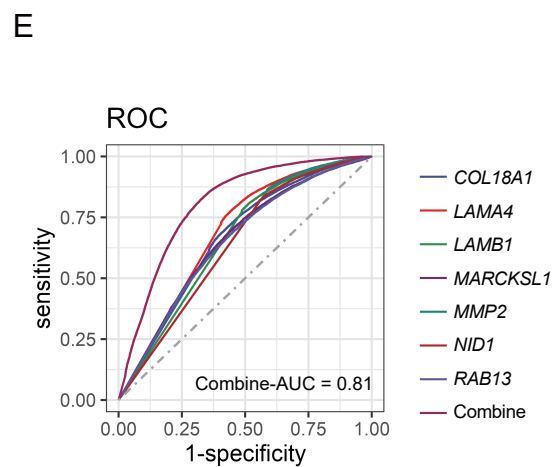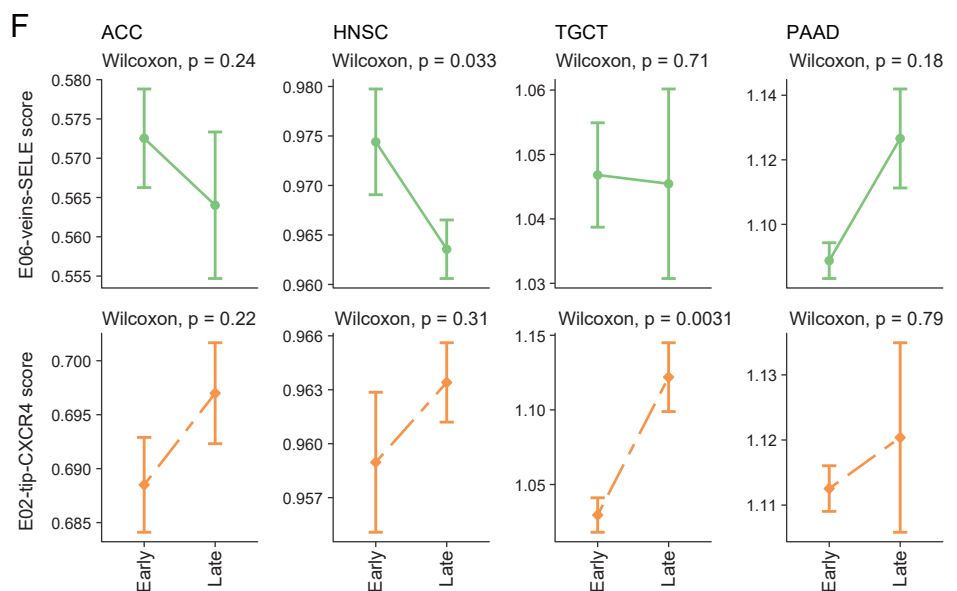

Supplement: nwae231_Supplemental_Files [file nwae231_supplemental_files.zip › Supplementary figures/sfig5.pdf]
